# Neuroligin 2 governs synaptic morphology and function through RACK1-cofilin signaling in *Drosophila*

**DOI:** 10.1038/s42003-023-05428-3

**Published:** 2023-10-18

**Authors:** Yichen Sun, Moyi Li, Junhua Geng, Sibie Meng, Renjun Tu, Yan Zhuang, Mingkuan Sun, Menglong Rui, Mengzhu Ou, Guangling Xing, Travis K. Johnson, Wei Xie

**Affiliations:** 1 School of Life Science and Technology, The Key Laboratory of Developmental Genes and Human Disease, Southeast University, Nanjing, 210096 China; 2 School of Biological Sciences, Monash University, Clayton, VIC 3800 Australia; 3 Jiangsu Co-innovation Center of Neuroregeneration, Nantong University, Nantong, 226001 China; 4 The Key Laboratory of Modern Toxicology, Ministry of Education, School of Public Health, Nanjing Medical University, Nanjing, 211166 China; 5 Department of Biochemistry and Chemistry, and La Trobe Institute for Molecular Science, La Trobe University, Bundoora, VIC 3086 Australia

**Keywords:** Neuroscience, Molecular neuroscience

## Abstract

Neuroligins are transmembrane cell adhesion proteins well-known for their genetic links to autism spectrum disorders. Neuroligins can function by regulating the actin cytoskeleton, however the factors and mechanisms involved are still largely unknown. Here, using the *Drosophila* neuromuscular junction as a model, we reveal that F-Actin assembly at the *Drosophila* NMJ is controlled through Cofilin signaling mediated by an interaction between DNlg2 and RACK1, factors not previously known to work together. The deletion of DNlg2 displays disrupted RACK1-Cofilin signaling pathway with diminished actin cytoskeleton proteo-stasis at the terminal of the NMJ, aberrant NMJ structure, reduced synaptic transmission, and abnormal locomotion at the third-instar larval stage. Overexpression of wildtype and activated Cofilin in muscles are sufficient to rescue the morphological and physiological defects in *dnlg2* mutants, while inactivated Cofilin is not. Since the DNlg2 paralog DNlg1 is known to regulate F-actin assembly mainly via a specific interaction with WAVE complex, our present work suggests that the orchestration of F-actin by Neuroligins is a diverse and complex process critical for neural connectivity.

## Abstract (web summary)

*Drosophila* Neuroligin2 promotes F-actin assembly through the RACK1-Cofilin signaling pathway to regulate synaptic morphology and function at neuromuscular junctions.

## Introduction

Synapses, the sites of communication between neurons and their targets, are in some respects specialized variants of the cell-cell junctions formed by other cell types^1^. The highly specialized neuromuscular junction (NMJ) is one of the well-studied synapses^2^. At these synapses, direct linkage via transmembrane adhesion molecules plays critical roles in synaptic development, formation, maturation and maintenance^3^. Neuroligins are single-pass transmembrane postsynaptic adhesion molecules involved in synaptic formation and function^4–22^. In recent years, Neuroligins and their binding partners the Neurexins have captured wide attention due to their potent synaptogenic properties and genetic association with autism spectrum disorder (ASD), a developmental neurological disorder^23^. *Drosophila* has four *neuroligin* genes (*dnlg1–4*), which have a close evolutionary relationship to their vertebrate homologs^7^. Due to their multiple, functionally redundant family members, there are still obstacles to directly assessing Neuroligins’ effects on synaptic formation. The current data from both mammals and flies strongly support the vital participation of Neuroligins in synaptic function and the maturation of the postsynaptic apparatus^7,14,19,22,24–32^. However, the delicate molecular mechanisms by which Neuroligins regulate these processes are not fully understood.

Previously, using the *Drosophila* NMJ as a model, we and others showed that all four *Drosophila* Neuroligins (DNlgs) play roles in synaptic formation and function, including the regulation of bouton growth, subsynaptic reticulum assembly, glutamate receptor (GluR) recruitment, and synaptic transmission^7,14,19,22,27–33^. In a recent study, we found that both DNlg1 and DNlg2 have a positive effect on filamentous actin (F-actin) polymerization, while DNlg1, but not DNlg2, directly interacts with the WAVE regulatory complex (WRC) via its C-terminal interacting sequence to organize postsynaptic F-actin assembly, and thus regulate synaptic structure and function at the NMJ^19^. This suggests that DNlg2 has a positive effect on F-actin polymerization via an unknown WRC-independent pathway.

F-actin polymerization is thought to be mainly mediated by WAVE-Arp2/3 pathway positively and by Cofilin negatively^34–37^. Hence, a potential non WAVE factor candidate is via Cofilin^38^, an actin binding protein abundantly expressed at the synaptic level that promotes F-actin depolymerization by both filament severing and monomeric actin (G-actin) dissociation from the pointed ends^38,39^. The depolymerizing function of Cofilin is reported to promote a high rate of actin treadmilling, which occurs when G-actin depolymerized from the pointed ends of the filaments is continuously polymerized onto their barbed ends, allowing continuous and robust actin structural reorganization to take place in dynamic cellular regions including for cytokinesis, axon growth and endocytosis^39–41^. All eukaryotes express at least one member of the essential actin-depolymerizing factor (ADF)/Cofilin family of actin-binding proteins^42^. In *Drosophila*, the gene *twinstar* (*tsr*) encodes the sole Cofilin homolog, which is highly conserved across all eukaryotes, from yeast and plants to mammals^43^. Loss of *tsr* causes multiple defects in cytokinesis, cell motility, axon growth, planar cell polarity and photoreceptor morphogenesis^42,44^. Loss of Cofilin in the murine nervous system leads to various synaptic and developmental defects and behavioral aberrance^43,45^. Neuronal cytoplasmic rods, which are abnormal hyperactive Cofilin-mediated F-actin aggregates^45^, accumulate within neurites, where they occlude neurites and block vesicle transport, and are a likely cause of synaptic loss without neuronal loss^45^. These synaptic dysfunctions may play a role in cognitive dementias and Alzheimer disease^45,46^. In this respect, Cofilin is considered a homeostatic regulator in cell biology^38^.

In this study, through immunoprecipitation (IP) and mass spectrometry (MS), we surprisingly uncover a direct interaction between DNlg2 and Receptor for activated C kinase 1 (RACK1, also known as Gnb2l1 in mammals), an evolutionarily conserved scaffolding protein that interacts with multiple signaling molecules concurrently through its seven Trp-Asp 40 (WD40) repeats^47–49^. Based on its wide variety of protein partners, RACK1 has been reported to play a role in diverse processes^47–49^. Germline deletion of RACK1 in *Drosophila* or mice causes embryonic arrest^48,49^. Beyond these, RACK1 has been reported to be a Cofilin regulator by a genome-wide RNA interference (RNAi) screen in cultured *Drosophila* S2R+ cells^50^. It is also known to directly interact with Rac1^51,52^ and RhoA^53^, which are two small GTPases involved in actin skeleton regulation.

Here, we reveal a significant role of DNlg2 in the regulation of the actin cytoskeleton in the postsynaptic NMJ through the RACK1-Cofilin signaling pathway. We find that the dramatic reduction in the amount of F-actin at NMJ in *dnlg2* mutants is due to the quantity imbalance between phosphorylated and non-phosphorylated forms of Cofilin. Wildtype and non-phosphorylated forms of Cofilin can reverse NMJ synapse undergrowth and reduce locomotion capability in *dnlg2* mutants, while a phospho-mimetic form of Cofilin cannot. The direct interaction between DNlg2 and RACK1 enriches our understanding of the RACK1 function on neuronal system and actin cytoskeleton organization. This study provides insights into the mechanisms by which Neuroligins regulate synaptic formation and function through diverse F-actin regulatory pathways.

## Results

### Loss of DNlg2 and reduced Cofilin cause similarly dysregulated F-actin assembly and synapse establishment

Previously it was shown DNlg2 has a positive effect on F-actin assembly similar to that of DNlg1^19^. However, the lack of interaction between DNlg2 and the WRC suggested it regulates postsynaptic F-actin assembly via a distinct mechanism to DNlg1. One possibility was that DNlg2 regulates F-actin via the Cofilin pathway because WRC and Cofilin signaling pathways are the two main mechanisms known to regulate F-actin dynamics^34–38^. Additionally, using a low-level ubiquitous driver (*da-GAL4*), we expressed DNlg2 tagged with EGFP and IP DNlg2-EGFP complexes for MS. The lysates identified several partner proteins of interest, including Cofilin (MS-based data can be found in Data Availability).

**Fig. 1:**
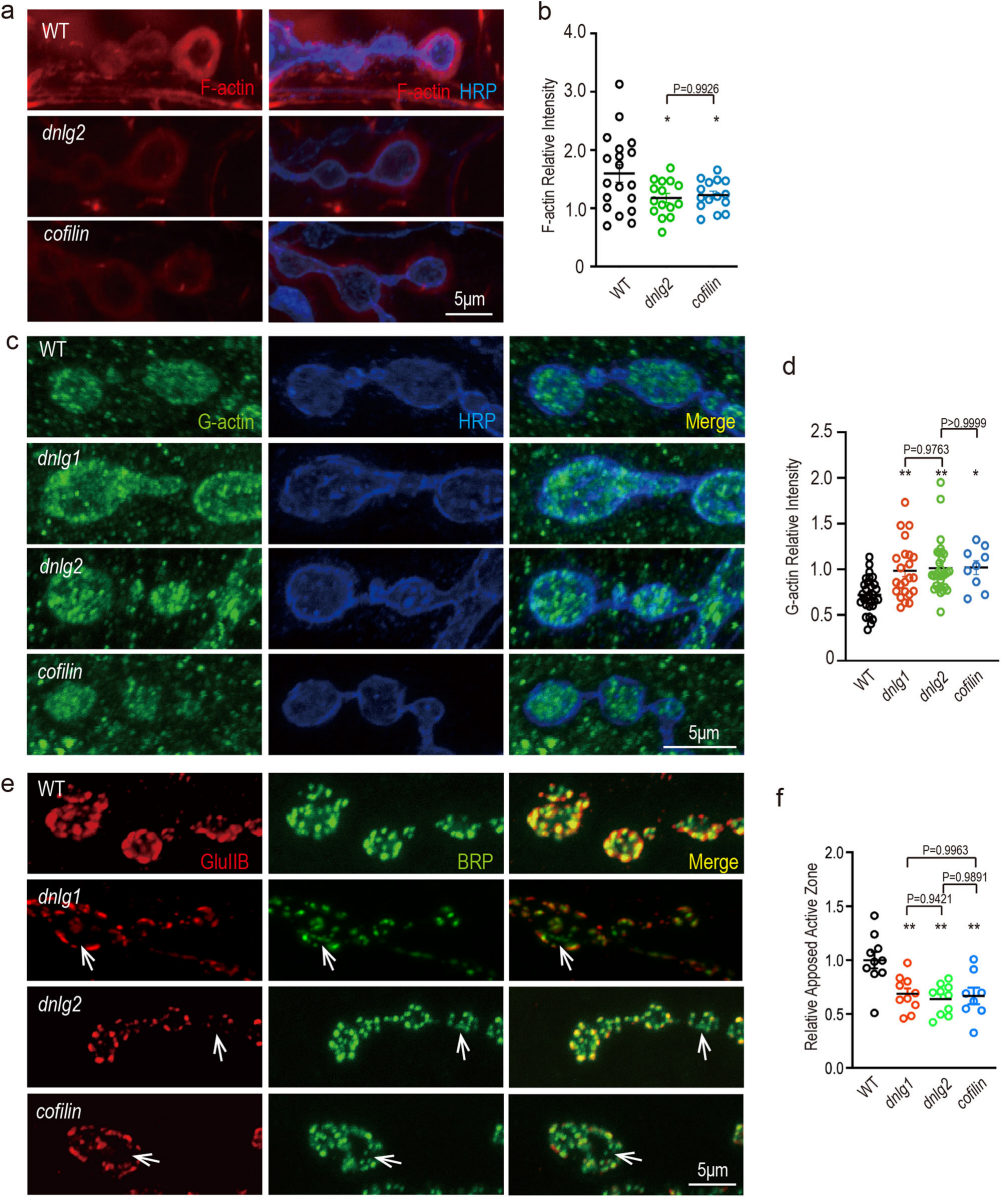
Actin dynamics and pre- and postsynaptic protein apposition are mis-regulated in *dnlg2* mutant NMJs. **a** F-actin is downregulated in *dnlg2* and *cofilin* mutants. Confocal images of WT (*n* = 19), *dnlg2* mutants (*dnlg2*^*KO70/KO70*^) (*n* = 15), and *cofilin* mutants (*tsr*^*N96A*^*/+*) (*n* = 14) third instar larvae NMJ type Ib boutons at muscles 12/13 labeled with Texas Red phalloidin (red, F-actin) and anti-HRP (blue). **b** Scatter diagram shows a significant decrease in the relative intensity of F-actin in *dnlg2* mutants, and *cofilin* mutants compared with WT. **c** G-actin is upregulated in *dnlg2* and *cofilin* mutants. Confocal images of WT (*n* = 29), *dnlg1* (*dnlg1*^*ex1.9/ex2.3*^) mutants (*n* = 23), *dnlg2* mutants (*n* = 30), and *cofilin* mutants (*n* = 9) third instar larvae NMJ type Ib boutons at muscles 12/13 labeled with anti-DNase I (green, G-actin) and anti-HRP (blue). **d** Scatter diagram shows a significant increase in the relative intensity of G-actin in *dnlg1* mutants, *dnlg2* mutants, and *cofilin* mutants compared with WT. **e** Relative apposed active zones decrease in *dnlg2* and *cofilin* mutants. Confocal images of WT (*n* = 10), *dnlg1* mutants (*n* = 10), *dnlg2* mutants (*n* = 10) and *cofilin* mutants (*n* = 8), third instar larvae NMJ type Ib boutons at muscle 4 labeled with anti-GluRIIB (red) and anti-nc82 (green, BRP). White arrowheads highlight the zones that GluRIIB cannot correspond to BRP. **f** Scatter diagram shows a significant decrease in the relative apposed active zone in *dnlg1* mutants, *dnlg2* mutants, and *cofilin* mutants compared with WT. Data are presented as mean ± SEM.

To test whether DNlg2 regulates F-actin through Cofilin at the NMJ, we first used an antibody (CF1) raised against Cofilin to examine its localization in wildtype flies. The *Drosophila* body-wall muscles are innervated by numerous motor neurons that branch over the muscles and form stereotypic NMJ terminal boutons^54^. Our immunostaining revealed Cofilin along the NMJ and the muscle, suggesting that it may have a local function at NMJ (Supplementary Fig. 1a). Next, we asked whether Cofilin influences F-actin distribution at the NMJ. Hence, a Cofilin null mutant, *tsr*^*N96A*^*/+*^55^, was used. Consistent with previous studies^19^, F-actin was highly enriched at the postsynaptic sites of the wildtype NMJ, displaying a diffuse meshwork-like appearance. In the *dnlg*2 and *cofilin* mutant NMJs, however, the amount of F-actin was dramatically reduced (Fig. 1a, b). Since Cofilin promotes the maintenance of the large G-actin pool by providing new actin monomers^56^, we also tested the amount of G-actin at the NMJ. In contrast to F-actin and consistent with our expectations, *dnlg2* and *cofilin* mutant NMJs had a significantly increased amount of G-actin compared to WT (Fig. 1c, d). These data suggest that DNlg2 and Cofilin are important for the actin cytoskeleton at the *Drosophila* NMJ.

As each bouton contains dozens of glutamatergic synapses^54^ and postsynaptic F-actin is important for GluRs recruitment and thus proper synapse establishment^57^, we therefore investigated the apposition between postsynaptic GluRs and presynaptic neurotransmitter release sites (active zones). In mutants for DNlg2 and Cofilin, approximately a third of presynaptic active zones were not aligned well with postsynaptic GluRs, while these two markers were mostly apposed in WT (Fig. 1e, f). These results indicate that DNlg2 and Cofilin are necessary for proper NMJ synapse establishment.

### DNlg2 regulates postsynaptic F-actin via Cofilin at the NMJ

**Fig. 2:**
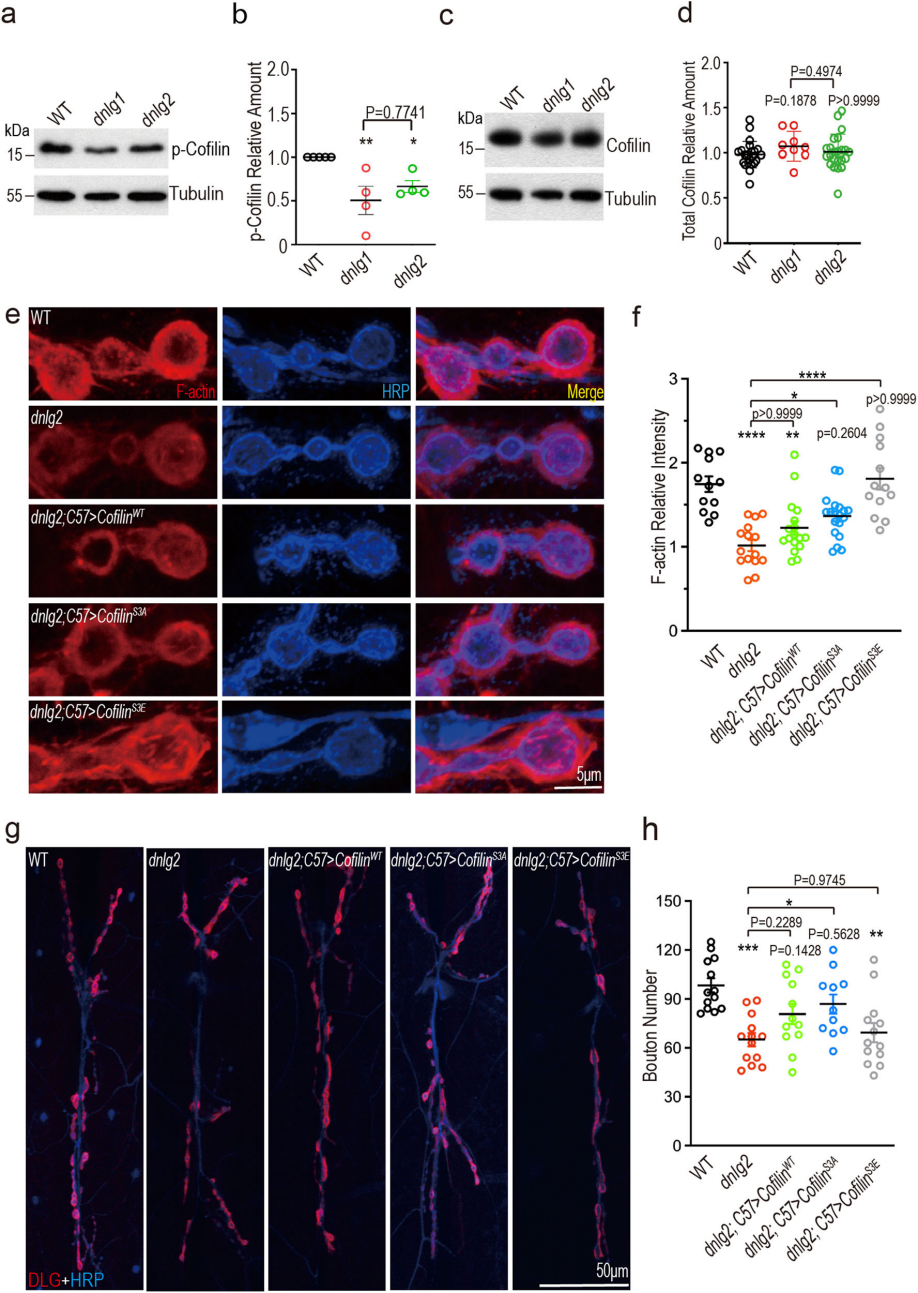
The decreased F-actin level and bouton number at the NMJ of *dnlg2* mutants could be rescued by modulating postsynaptic Cofilin activity. **a** p-Cofilin is downregulated in *dnlg2* mutants. Muscle lysates of the third instar larvae from WT (*n* = 5), *dnlg1* mutants (*n* = 4), and *dnlg2* mutants (*n* = 4) were subjected to western blots with anti-p-Cofilin antibody. **b** Scatter diagram shows the relative amount of p-Cofilin in both lines in **a**. p-Cofilin expression was dramatically inhibited in *dnlg1* mutant and *dnlg2* mutant. **c** Muscle lysates of the third instar larvae from WT (*n* = 23), *dnlg1* mutants (*n* = 9), and *dnlg2* mutants (*n* = 23) were subjected to western blots with anti-Cofilin antibody. **d** Scatter diagram of the total Cofilin relative amount in **c**. There was no change in the total Cofilin expression in *dnlg1* mutants and *dnlg2* mutants. **e** Confocal images of the third instar larvae NMJs from WT (*n* = 12), *dnlg2* mutants (*n* = 15), *dnlg2; C57* *>* *Cofilin*^*WT*^ (*n* = 17), *dnlg2; C57* *>* *Cofilin*^*S3A*^ (*n* = 19) and *dnlg2; C57* *>* *Cofilin*^*S3E*^ (*n* = 13). Type Ib boutons at muscles 12/13 were labeled with Texas Red phalloidin (red) and anti-HRP (blue). **f** Quantitative data of **e** shows the decreased F-actin relative intensity in *dnlg2* mutants and restoration by muscle expression of Cofilin. **g** Confocal images of the third instar larvae NMJs from WT (*n* = 13), *dnlg2* mutants (*n* = 13), *dnlg2; C57* *>* *Cofilin*^*WT*^ (*n* = 12), *dnlg2; C57* *>* *Cofilin*^*S3A*^ (*n* = 11) and *dnlg2; C57* *>* *Cofilin*^*S3E*^ (*n* = 13). Type Ib boutons at muscles 6/7 were labeled with anti-DLG (red) and anti-HRP (blue). **h** Quantitative data of **g** shows the reduced bouton number in *dnlg2* mutants and restoration by muscle expression of Cofilin. Data are presented as mean ± SEM.

Since the *dnlg2* and *cofilin* mutants both showed similar defects in postsynaptic actin organization and GluRs recruitment, we hypothesized that DNlg2 and Cofilin may act together to coordinate NMJ F-actin assembly. Though a previous study showed DNlg1 regulates F-actin through the interaction with WRC, we included *dnlg1* mutants in our tests of the Cofilin pathway. Specifically, we measured the phosphorylated, inactive form of Cofilin, as well as total Cofilin in WT, *dnlg1* and *dnlg2* mutant adult head and third instar larvae body-wall extracts by immunoblots. Both *dnlg* mutants showed a significant decrease in p-Cofilin level compared to WT (Fig. 2a, b), with no change in total Cofilin (Fig. 2c, d), suggesting that the loss of DNlg1/2 may cause a net increase in Cofilin activity. While we detected a disruption of p-Cofilin in *dnlg1*, hereon we focused our attention on DNlg2. In this regard, these data raise the possibility that DNlg2 promotes postsynaptic F-actin assembly at the NMJ via the modulation of Cofilin.

We reasoned that if DNlg2 acts upstream of Cofilin, then the defects caused by loss of Dnlg2 might be rescued by modulating postsynaptic Cofilin activity. Since Cofilin plays the dual functions to promote F-actin disassembly by severing, and polymerization by supplying actin monomers, we were unsure whether increasing or decreasing Cofilin activity would help actin reorganization and rescue the defects in *dnlg2* mutants^43^. For this reason, we started with overexpression of wildtype (Cofilin^WT^), constitutively active (Cofilin^S3A^, which cannot be phosphorylated), and constitutively inactive (Cofilin^S3E^, which mimics p-Cofilin^58^) Cofilin with a muscle-specific driver (*C57-GAL4*) under the *dnlg2* mutant background.

In the terms of the declined F-actin level at *dnlg2* mutants NMJ, postsynaptic overexpression of Cofilin^WT^ had no significant rescue effect but a restoration trend (Fig. 2e, f). Indeed, both Cofilin^S3A^ and Cofilin^S3E^ could rescue this deficit, with a stronger rescue effect by Cofilin^S3E^ (Fig. 2e, f). However, unlike the wildtype NMJ, in which F-actin organization is evenly spaced and distributed around the bouton (Fig. 2e), F-actin in the *dnlg2* mutants expressing Cofilin^S3E^ was organized into snarls and rods, which was visibly abnormal (Fig. 2e). We were therefore curious as to whether other *dnlg2* NMJ deficits could also be rescued.

As a measure of the NMJ growth, bouton number in *dnlg2* mutants was previously found declined^28,59^. While expression of Cofilin^WT^ partially increased the bouton number in *dnlg2* mutants, expression of Cofilin^S3A^ did so significantly and to a larger degree relative to the *dnlg2* mutant control (Fig. 2g, h). Bouton number in the Cofilin^S3E^ rescue line was significantly lower than that of wildtype and comparable to that of *dnlg2* mutants (Fig. 2g, h).

**Fig. 3:**
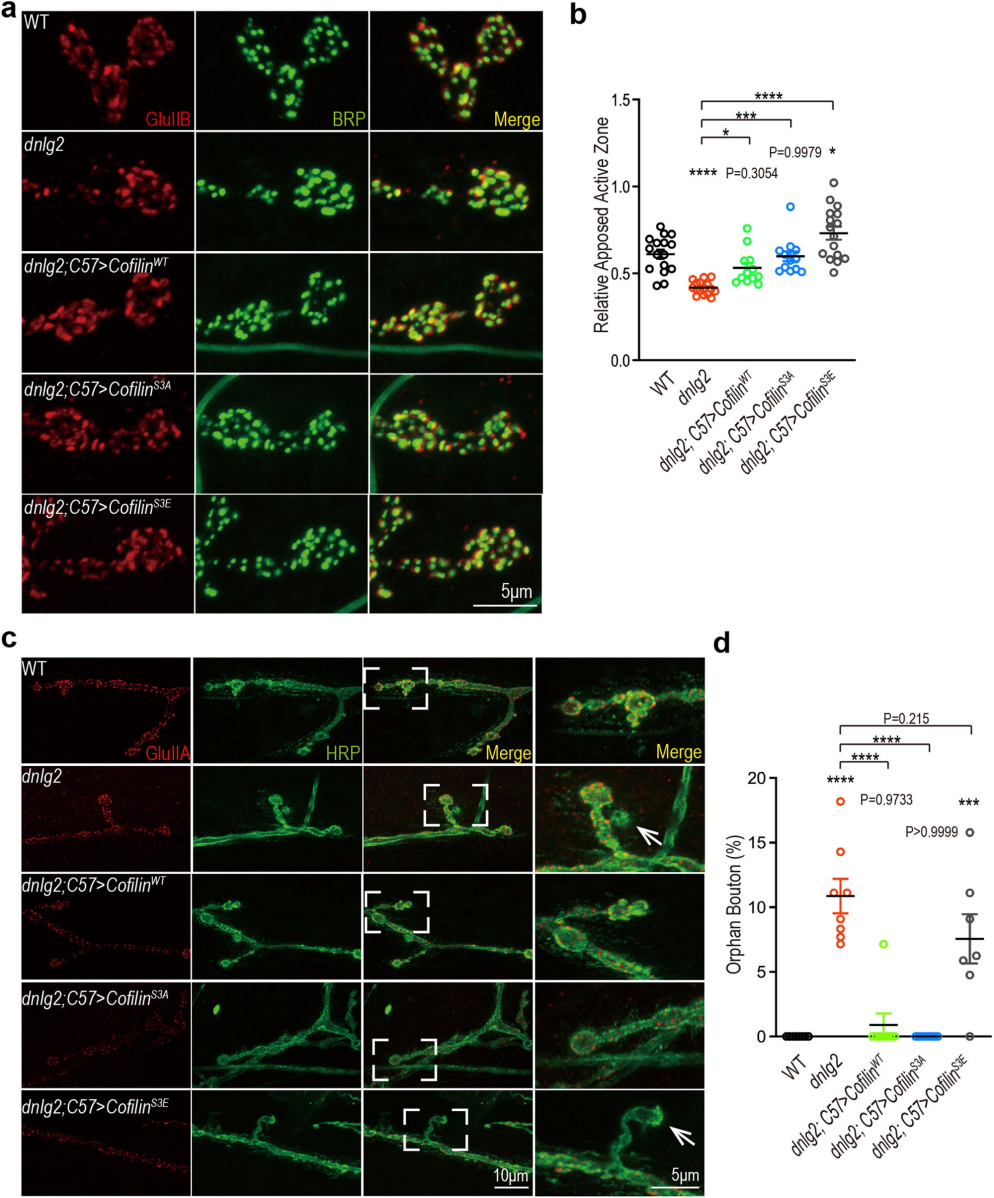
The aberrant GluRs recruitment at the NMJ of *dnlg2* mutants could be rescued by modulating postsynaptic Cofilin activity. **a** Confocal images of the third instar larvae NMJs from WT (*n* = 16), *dnlg2* mutants (*n* = 16), *dnlg2; C57* *>* *Cofilin*^*WT*^ (*n* = 12), *dnlg2; C57* *>* *Cofilin*^*S3A*^ (*n* = 13) and *dnlg2; C57* *>* *Cofilin*^*S3E*^ (*n* = 16). Type Ib boutons at muscle 4 were labeled with anti-GluRIIB (red) and anti-nc82 (green, BRP). **b** Quantitative data of **a** shows the decreased apposed active zone in *dnlg2* mutants and restoration by muscle expression of Cofilin^WT^, and Cofilin^S3A^. Cofilin^S3E^ over-rescued this defect. **c** Confocal images of the third instar larvae NMJs from WT (*n* = 8), *dnlg2* mutants (*n* = 8), *dnlg2; C57* *>* *Cofilin*^*WT*^ (*n* = 8), *dnlg2; C57* *>* *Cofilin*^*S3A*^ (*n* = 8) and *dnlg2; C57* *>* *Cofilin*^*S3E*^ (*n* = 7). Type Ib boutons at muscle 4 were labeled with anti-8B4D2 (red, GluRIIA) and anti-HRP (blue). The right lane shows the amplified contents framed in merge images. White arrowheads highlight the orphan boutons. **d** Quantitative data of **c** shows the increased percentage of orphan bouton in *dnlg2* mutants and restoration by muscle expression of Cofilin^WT^ and Cofilin^S3A^. Data are presented as mean ± SEM.

We next looked at synapse establishment in these NMJs and found the aberrant GluRs recruitment in *dnlg2* mutants was strongly restored by expression of wildtype and constitutively active Cofilin (Fig. 3a, b). However, interestingly, Cofilin^S3E^ had a much more pronounced rescue effect on the mis-apposition phenotype in *dnlg2* mutants, causing rescue of the phenotype to a level even beyond the wildtype control (Fig. 3a, b). We also noticed orphan boutons^29^, a poor synaptic development phenotype, in the *dnlg2* mutants whereby around 10% boutons lost the postsynaptic receptors (Fig. 3c, d). Both Cofilin^WT^ and Cofilin^S3A^ completely rescued this defect in *dnlg2* mutants, whereas Cofilin^S3E^ could not (Fig. 3c, d).

**Fig. 4:**
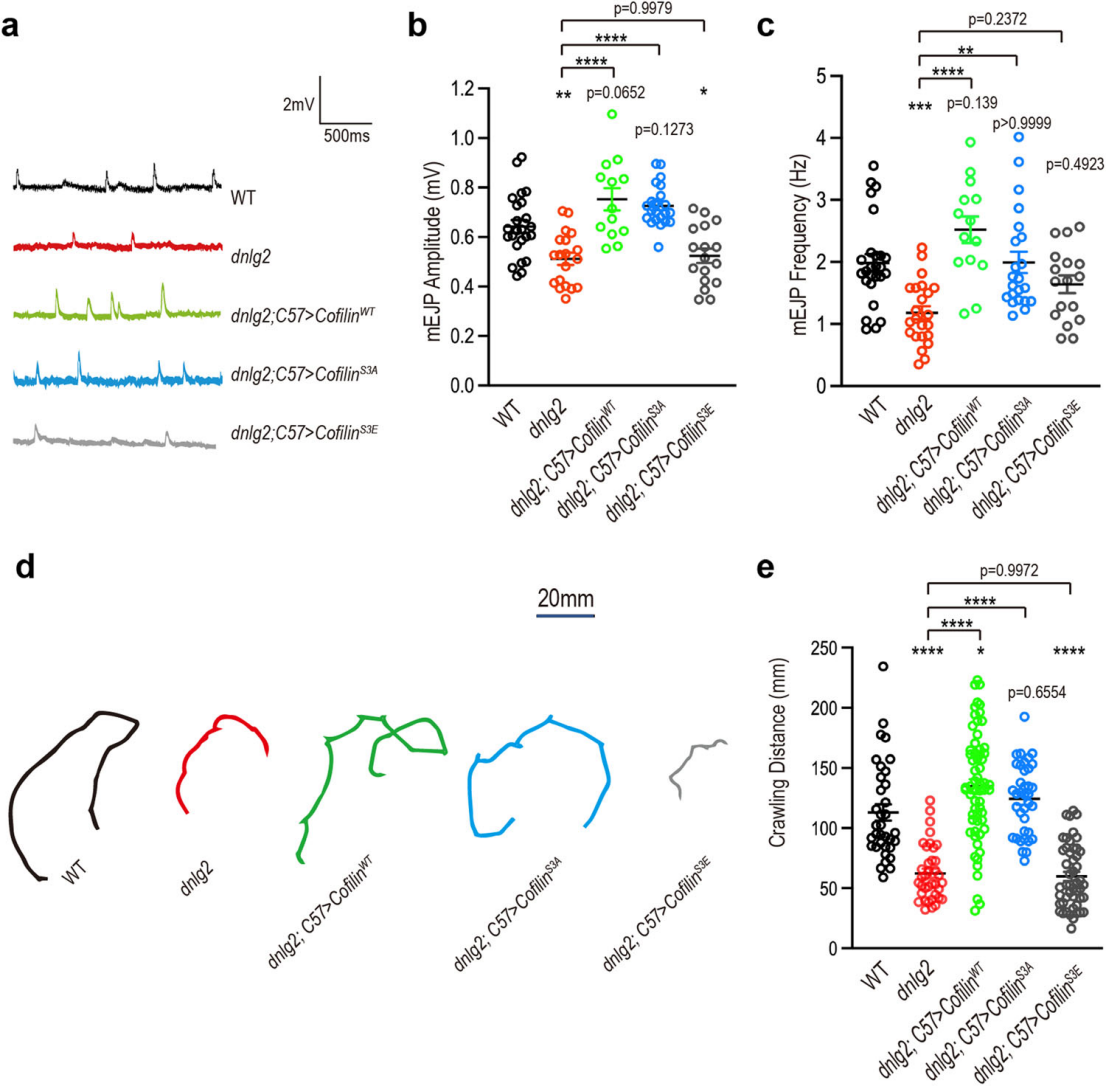
The aberrant synaptic transmission and locomotion activity in *dnlg2* mutants could be rescued by modulating postsynaptic Cofilin activity. **a** Representative trace of the mEJP. **b** Scatter diagram of the mean mEJP amplitude (in mV) from WT (*n* = 24), *dnlg2* mutants (*n* = 19), *dnlg2; C57* *>* *Cofilin*^*WT*^ (*n* = 13), *dnlg2; C57* *>* *Cofilin*^*S3A*^ (*n* = 22) and *dnlg2; C57* *>* *Cofilin*^*S3E*^ (*n* = 17) third instar larvae. **c** Scatter diagram of the mean mEJP frequency (in Hz) from WT (*n* = 26), *dnlg2* mutants (*n* = 23), *dnlg2; C57* *>* *Cofilin*^*WT*^ (*n* = 14), *dnlg2; C57* *>* *Cofilin*^*S3A*^ (*n* = 22) and *dnlg2; C57* *>* *Cofilin*^*S3E*^ (*n* = 17) third instar larvae. **d** Representative traces of a 3-min crawling of WT (total *n* = 35), *dnlg2* mutants (*n* = 37), *dnlg2; C57* *>* *Cofilin*^*WT*^ (*n* = 63), *dnlg2; C57* *>* *Cofilin*^*S3A*^ (*n* = 37) and *dnlg2; C57* *>* *Cofilin*^*S3E*^ (*n* = 49) third instar larvae. **e** Quantification of a 3-min crawling distance (in mm) in **d**, showing a reduced locomotor activity in *dnlg2* mutants and restoration by muscle expression of Cofilin^WT^ and Cofilin^S3A^. Data are presented as mean ± SEM.

As the stability and integrity of synapses are vital for the maintenance of mature synapse function^60^, we further analyzed the synaptic transmission ability and animal behavior of the third-instar larvae. In line with previous studies^28^, synaptic transmission capacity was decreased in *dnlg2* mutants, as we detected the declined mEJP amplitude and mEJP frequency (Fig. 4a–c). Postsynaptic expression of Cofilin^WT^ and Cofilin^S3A^ in the *dnlg2* mutants restored the mEJP amplitude back to levels comparable to those in WT, whereas Cofilin^S3E^ did not (Fig. 4a–c). Intriguingly, the mEJP frequency could be rescued by all three Cofilin lines, though Cofilin^S3E^ had the weakest rescue effect (Fig. 4a–c), suggesting the Cofilin^S3E^ might regulate synaptic transmission via alternative means. In the terms of locomotion, consistent with previous studies^14^, *dnlg2* mutants showed severe reduction in crawling ability, covering approximately half the distance of WT (Fig. 4d, e). Postsynaptic expression of Cofilin^WT^ and Cofilin^S3A^ in the *dnlg2* mutants rescued this defect back to levels comparable to that of WT, whereas Cofilin^S3E^ could not (Fig. 4d, e).

Taken together, these results suggest that increasing postsynaptic Cofilin activity buffers the impacts of DNlg2 loss through the maintenance of synaptic morphology and establishment, which in turn preserves normal synaptic transmission capacity and locomotor behavior. As Cofilin function is typically associated with the severing of F-actin rather than its reassembly, these data fit better under a model whereby DNlg2-Cofilin enhances the supply of G-actin monomer required for filament growth.

Additionally, these data also suggest that the NMJ morphological and functional defects observed upon Dnlg2 deficiency are the result of a dysregulation of F-actin assembly, caused by reduced Cofilin activity rather than merely insufficient F-actin levels, as the F-actin stabilization is not sufficient to restore synaptic growth, integrity, transmission, and locomotion.

### DNlg2 binds RACK1 and may regulate Cofilin activity via RACK1-mediated Rac1 and RhoA pathways

Having established the relationship between DNlg2 and Cofilin for regulating F-actin assembly at synapse, we next wished understand how DNlg2 signals to Cofilin activity.

**Fig. 5:**
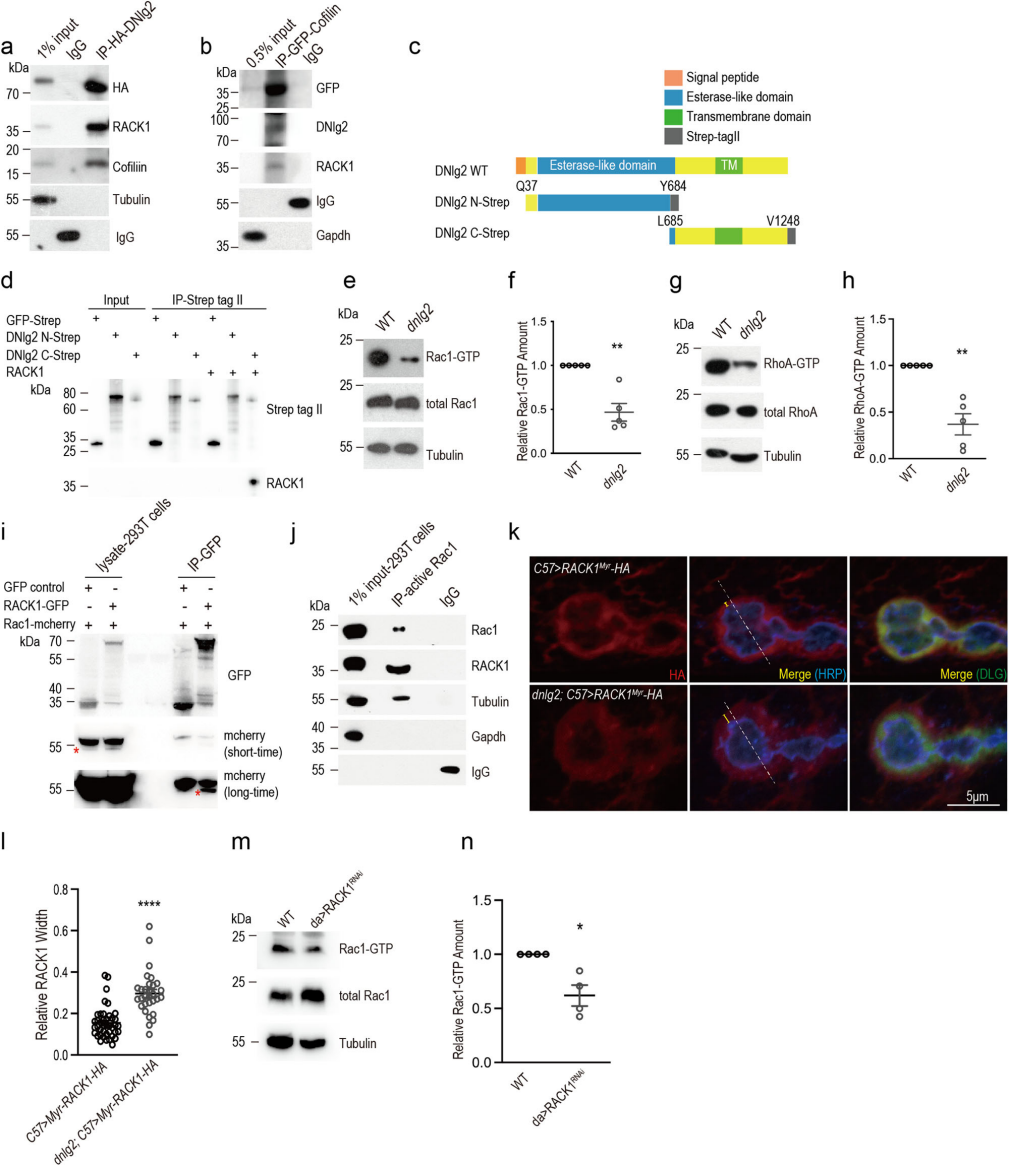
DNlg2 can interact with RACK1 and Cofilin, and RACK1 can potentially interact with active Rac1. **a** DNlg2, RACK1 and Cofilin can interact with each other in vivo. IP lysates was enriched from *da-GAL4* > *UAS-DNlg2-HA* adult brain extracts with anti-HA antibody. **b** DNlg2, RACK1 and Cofilin can interact with each other in vivo. IP lysates was enriched from *da-GAL4* > *UAS-Cofilin-GFP* adult brain extracts with anti-GFP antibody. **c** Schematic diagram of the synthesis N-terminal (Q37-Y684) and C-terminal (L685-V1248) fragments of DNlg2 bound to the Strep tag II. **d** DNlg2 binds RACK1 through its C-terminal fragment. Only the C-terminal of DNlg2 can directly bind to RACK1, whereas DNlg2 N-terminal cannot. **e** Western blots show the level of Rac1-GTP in adult heads of WT and *dnlg2* mutants. **f** Scatter diagram of the Rac1-GTP relative amount in **e**, indicating the decreased Rac1-GTP in *dnlg2* mutants. **g** Western blots show the level of RhoA-GTP and total RhoA in adult heads of WT and *dnlg2* mutants. **h** Scatter diagram of the RhoA-GTP relative amount in **g**, indicating the decreased RhoA-GTP in *dnlg2* mutants. **i** IP from HEK293T extracts with overexpression of *Drosophila* RACK1-GFP and Rac1-mcherry, enriched with GFP antibody. The result suggests *Drosophila* RACK1 physically interacts with *Drosophila* Rac1 in vitro. The asterisks indicate the proper bands. **j** Active Rac1 can interact with RACK1 in HEK293T cells. HEK293T cell lysates were subjected to a pull-down assay with GST-PAK-PBD agarose beads. **k** Confocal images of the third instar larvae NMJs from *C57>Myr-RACK1-HA* (*n* = 41) and *dnlg2; C57>Myr-RACK1-HA* (*n* = 30). Type Ib boutons at muscle 4 were labeled with anti-HA (red, tagged with RACK1^Myr^), anti-HRP (blue) and anti-DLG (green). **l** Quantitative data of **k** shows the relative RACK1 width on boutons. **m** Western blots show the level of Rac1-GTP in the third instar larvae NMJs from WT (*n* = 4) and *da* > *RACK1*^*RNAi*^ (*n* = 4). **n** Scatter diagram of the Rac1-GTP relative amount in *n*, indicating the decreased Rac1-GTP when knocking down RACK1. Data are presented as mean ± SEM.

To begin, we intended to identify proteins that complex with DNlg2, and may therefore be involved in signaling to Cofilin. In the MS results for DNlg2 IP, a well-known scaffold protein for signaling complexes, RACK1, was detected. To confirm the interactions between DNlg2, Cofilin and RACK1, we enriched DNlg2 complexes from DNlg2-HA-overexpressing (*da-GAL4*) *Drosophila* adult head extracts and performed co-IP analysis (Fig. 5a). Both RACK1 and Cofilin were detected (Fig. 5a). Similarly, DNlg2 and RACK1 were detected in the IP lysates from Cofilin-GFP-overexpressing (*da-GAL4*) *Drosophila* adult head tissue (Fig. 5b). We also validated the interaction between Nlg1 and RACK1 in mouse brain (Supplementary Fig. 2a). Mouse Nlg1 is the homolog of DNlg2 in *Drosophila* suggesting that the interaction between Nlgs and RACK1 is conserved through to mammals. These results strongly suggest that DNlg2 exists in complex with both RACK1 and Cofilin in vivo.

To further determine which part of DNlg2 binds RACK1 and Cofilin, we performed an additional MS on the extracellular and intracellular domains of DNlg2, respectively (MS-based data can be found in Data Availability). RACK1 was identified as an interactor of the DNlg2 intracellular domain, but not the extracellular domain (Table 1). Cofilin, however, was associated with both domains (Table 1). We also synthesized N- and C-terminal fragments of DNlg2 bound to the Strep tag II, respectively (Fig. 5c), and performed in vitro pull-down assays with synthesized *Drosophila* RACK1. Consistent with the MS results, only the C-terminal of DNlg2 was found to bind RACK1 (Fig. 5d). These combined results suggest the specific direct interaction between DNlg2 C-terminal fragment and RACK1, and provide a basis for the direct regulation of F-actin regulation to influence synapse morphology and function.

**Table 1:** Mass spectrometry analysis for the extra- and intracellular domains of DNlg2 GST pull down lysates from *Drosophila* adult heads.

MS detected protein	DNlg2		RACK1		Cofilin	
GST tagged domain	DNlg2-E	DNlg2-I	DNlg2-E	DNlg2-I	DNlg2-E	DNlg2-I
Pep Count^a^	211	0	0	6	20	17
Unique Pep Count^b^	18	0	0	6	5	9
Cover Percent^c^	18.67%	0	0	19.18%	39.19%	67.57%

E = extracellular domain (M1-S965).

I = intracellular domain (A989-V1248).

^a^The number of total detected peptide reads for a protein acquired in MS analysis.

^b^The number of unique peptides acquired from MS analysis.

^c^Coverage of peptide reads over the whole protein sequence.

As RACK1 has been linked to Cofilin phosphorylation previously^50,53^, we were curious to know whether the expression or localization of RACK1 is abnormal when DNlg2 is deficient, which in turn may affect Cofilin. Although no changes in the expression level of RACK1 were detected in *dnlg2* mutants and the striatum of Nlg1 knockout mice (Supplementary Fig. 2b–e), we found that RACK1 showed a diffused distribution at the NMJ of *dnlg2* mutants (Fig. 5k), suggesting a role of DNlg2 in regulating the localization of RACK1, which may further affect its function. We also detected an increase in total Cofilin levels when ubiquitously knocked down RACK1 by *da-Gal4* (Supplementary Fig. 2b–e).

Under the canonical model of Cofilin regulation, the G-protein Rac1 activates PAK1, a serine/threonine kinase that phosphorylates LIMK, which in turn phosphorylates Cofilin^58^. There is also an alternative pathway whereby LIMK is activated by RhoA-ROCK^58^. To see whether one or both of these pathways are working in DNlg2-Cofilin signaling, we looked at the activity of Rac1 and RhoA in *dnlg2* mutants using antibodies that detect GTP-bound (active) and their total protein level, respectively. We detected strong reduction in both activated Rac1 and RhoA in *dnlg2* mutants (Fig. 5e–h), which is consistent with the reduction of p-Cofilin detected in *dnlg2* mutants, suggesting DNlg2 may regulate the activity of Cofilin through Rac1 and RhoA.

**Fig. 6:**
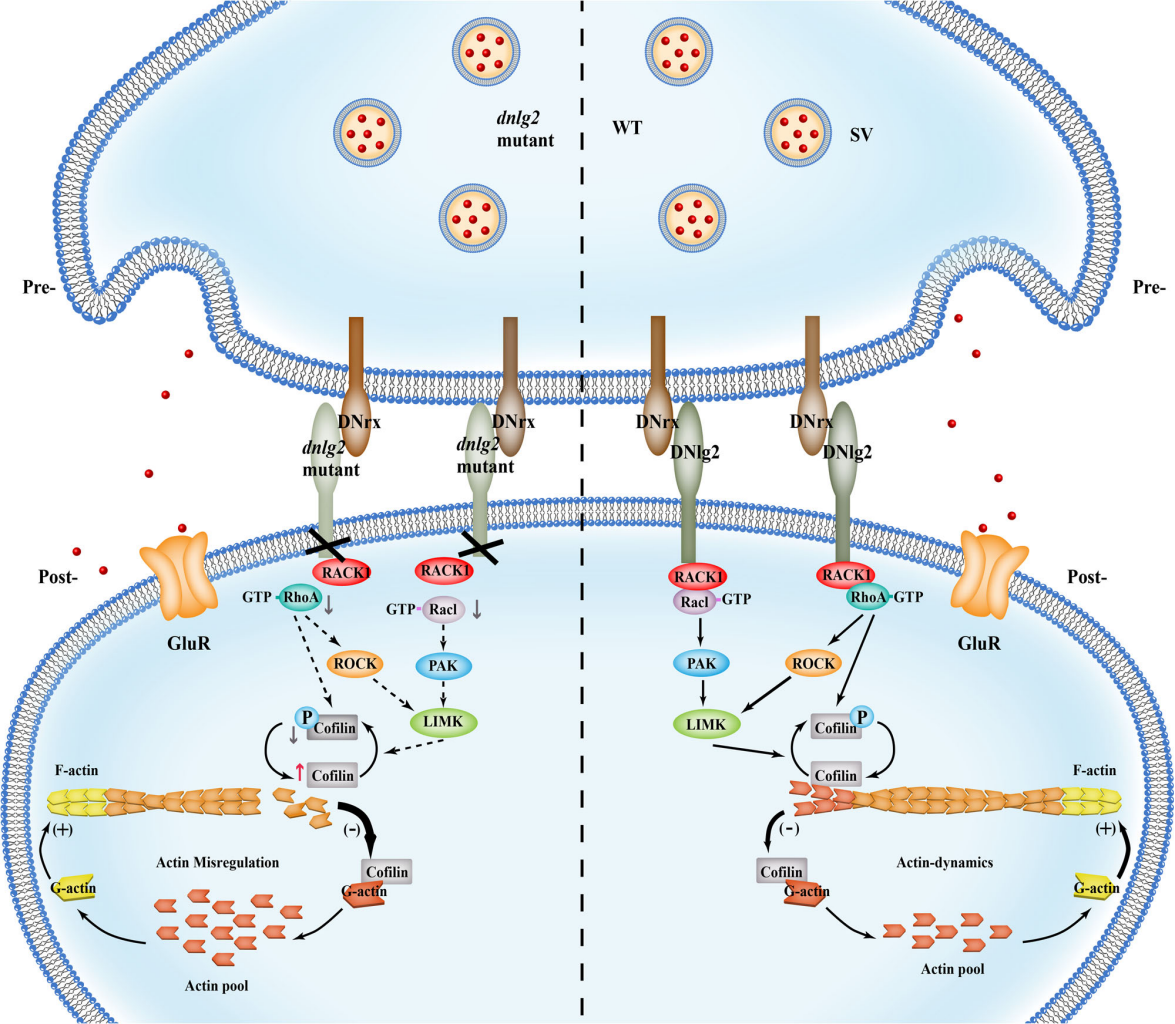
Model for Neuroligin2-RACK1-Cofilin signaling in WT and *dnlg2* mutants. In WT *Drosophila* NMJs, postsynaptic DNlg2 interacts with RACK1 which can form a complex with downstream Rac1-Cofilin or RhoA-Cofilin signaling components to activate Cofilin phosphorylation. Only if phosphorylated and non-phosphorylated Cofilin kept in a right balance, can actin polymerization/depolymerization be maintained in a good balance. Otherwise, disruption of DNlg2 will destabilize the big DNlg2-RACK1-Rac1 (or RhoA)-Cofilin-signaling complex. This disruption will affect Rac1 and RhoA activation and therefore inhibit downstream Cofilin phosphorylation. The downregulated p-Cofilin will break F-actin polymerization/depolymerization balance to accelerate its depolymerization.

Previous studies have identified RACK1 as an interactor of Rac1 in rice^51^, *C. elegans*^52^, and mouse^61^. We therefore hypothesized that RACK1 might similarly directly interact with Rac1 in *Drosophila*. To test this, we co-expressed GFP-tagged *Drosophila* RACK1 and mcherry-tagged *Drosophila* Rac1 in 293T cells and performed co-IP. We detected Rac1-mcherry only when co-expressing RACK1-GFP (Fig. 5i), suggesting that these proteins form a complex in vitro. Next, we enriched autologous active Rac1 in 293T cells and detected RACK1 in Rac1-GTP pull-down lysates (Fig. 5j). This validated the interaction between active Rac1 with RACK1 from 293T cells. According to bioinformatic analysis, the Rac1 and RACK1 protein sequences between *Drosophila* and human are highly similar (Supplementary Fig. 2h), and these two proteins have a high potential to form a complex in fly, as well as human and mouse (Supplementary Fig. 2i). Additionally, the expression level of activated Rac1 was reduced when knocking down RACK1 (Fig.5m, n), in line with our observations from *dnlg2* mutants, suggesting that the interaction between RACK1 and Rac1 is important for the activity of Rac1, and may further regulate Cofilin activation. Taken together these results support a model for DNlg2 regulation of Cofilin via DNlg2-RACK1-RhoA and DNlg2-RACK1-Rac1 pathways (Fig. 6).

## Discussion

Neuroligins affect synaptic development and functions largely through regulating F-actin assembly. Previously, we found DNlg1 can directly interact with WRC to promote F-actin polymerization through WRC-Arp2/3 signaling^19^. We observed similar F-actin downregulation in *dnlg2* mutants but it remained unknown how DNlg2 regulated F-actin organization, as DNlg2 does not bind directly with WRC. In this study, we detected p-Cofilin downregulation and G-actin promotion in *dnlg2* mutants, which suggests that the depolarization of F-actin is accelerated in *dnlg2* mutants. Then we observed most of F-actin related NMJ defects can be rescued by wildtype and activated forms of Cofilin. Activated Rac1 and RhoA, the positive regulators of Cofilin phosphorylation are dramatically downregulated in *dnlg2* mutants at the same time, thus, suggesting the Cofilin phosphorylation pathway is disrupted. The reduced Rac1 and RhoA activation in *dnlg2* mutants is likely to cause a major imbalance between activated and inactivated Cofilin, ultimately resulting in accelerated F-actin depolymerization.

How can DNlg2 suppress Rac1 and RhoA activation and further suppress Cofilin phosphorylation? Our MS and co-IP experiments fished out an actin related regulator, RACK1 in the complex with DNlg2, and the further MS analysis indicates that RACK1 specifically interacts with DNlg2 intracellular domain instead of extracellular domain, whereas Cofilin does not display this unique feature. These finding highly suggest that the C-terminal of DNlg2 can directly interact with RACK1 to connect DNlg2 and small G-proteins regulating Cofilin activity, as shown in Fig. 6.

RACK1 has gained recent attention in neuroscience due to its rediscovered potential functions in neural systems^62^. It is reported to be involved in axon growth and guidance in *C. elegans*^52^. Additionally, it can act as the regulator of the actin cytoskeleton in migrating cells and growth cones^63,64^. More recent studies found its role in mouse cerebellar and cortex development through regulating multiple different pathways in neural stem cells^65,66^. Our observation that knocking down RACK1 reduced active Rac1 might be a hint of how RACK1 functions, because Rac1 appears to regulate a diverse array of cellular events^58,67–70^. We also found a conserved interaction between Nlg1 and RACK1 in mouse brain tissue, and it would be interesting to test whether Nlg1 functions through RACK1 in mammalian neural system.

An unexpected finding was that DNlg2 deletion had no discernible effect on RACK1 accumulation, or total Rac1 and RhoA in cells. It seems that DNlg2 deletion only affects the distribution of RACK1, destabilizing RACK1-Rac1-Cofillin or RACK1-RhoA-Cofillin complexes, and therefore affecting Rac1 or RhoA activation. Surprisingly, although most *dnlg2* defects could only be restored by Cofilin^WT^ and Cofilin^S3A^, Cofilin^S3E^ rescued F-actin, apposed active zones, and the mEJP frequency in *dnlg2* mutants. These restoration effects might due to the remaining F-actin depolymerizing activity in Cofilin^S3E^, which is about 10% of the wildtype Cofilin^71^. However, it is still puzzling why Cofilin^S3E^ had a stronger rescue effect than Cofilin^WT^ and Cofilin^S3A^ on F-actin or apposed active zone rescue experiments. These suggest the transition between activated and inactivated Cofilin is more complicated than our expectation, and thus requires further investigation.

The *dnlg1* mutant also displayed disrupted p-Cofilin accumulation. This result is in contrast with our original hypothesis that DNlg1 and DNlg2 may have distinct F-actin regulation pathways. F-actin organization is rescued by recovering the interaction of DNlg1 and WRC^10^ in *dnlg1* mutants, so it remains to be known how the observed p-Cofilin down-regulation contributes to *dnlg1* phenotypes. One possibility is that DNlg1 regulates F-actin assembly primarily via the WAVE complex but also utilizes Cofilin. It would be interesting see if Cofilin^WT^, Cofilin^S3A^ or Cofilin^S3E^ can partially reverse *dnlg1* phenotypes.

There are two main limitations to this study. First, despite we detected the disorganized F-actin like snarls and rods in the *dnlg2* mutants expressing Cofilin^S3E^ by confocal, a higher resolution imaging modality might be able to better resolve the disorganized F-actin, and to investigate the difference compared to the evenly spaced and distributed F-actin organization. Second, we showed that RACK1 can directly interact with and regulate Rac1 activity. While we would like to confirm this via other approaches, unfortunately, there is a lack of effective immunofluorescence staining antibodies to detect the in vivo Rac1. Development of such reagents would enable the localization of Rac1 in RACK1 knockout or RNAi *Drosophila* lines.

Taken together, we discover a previously unknown DNlg2-RACK1-Cofilin signaling pathway to suppress the F-actin depolymerization process. This is different to how DNlg1 primarily regulates synaptic morphology and function through WRC in *Drosophila* NMJ. These diverse F-actin assembly regulatory mechanisms of Neuroligins at the *Drosophila* NMJ suggests the potential for similar mechanisms present in other higher animals and even in humans. These findings provide unique insights to further understand the basic working principles behind normal neural functions and molecular pathogenesis of neural disorders like ASD.

## Methods

### Fly stocks

All flies were reared at 25 °C in standard medium. The *w*^*1118*^ flies were used as WT controls in this study. The following fly mutants were used: *dnlg1* mutant: *dnlg1*^*ex1.9/ex2.3*^, a heterozygous mutant combined with two excision alleles^7^; *dnlg2* mutant: *dnlg2*^*KO70*^ generated by our lab, which lost the first exon as well as the expression of DNlg2^14^; *cofilin* mutant: *tsr*^*N96A*^*/+* (BDSC, 9108). *tsr*^*N96A*^ is a null allele, which has a 676 bp deletion in the 5′ region including the first exon, consistent with a functional loss of Cofilin^55^. As its homozygous mutant is lethal before the end of first instar larval stage, heterozygous mutant was used here. *rack1* mutant: *rack1*^*EY00128/1.8*^, a heterozygous mutant combined with two null alleles (BDSC, 15000 and 24152); RACK1^RNAi^ (VDRC, V27858). *UAS-Myr-RACK1-HA* is a membrane-tethered form of RACK1 by adding a myristoylation signal at its N-terminus^72^. The muscle-specific driver *C57-Gal4* and systemic driver *da-Gal4* were obtained from BDSC.

Three muscle-specific rescue lines were crossed as:

*dnlg2* ^*KO70*^ *; C57-GAL4 > UAS-Cofilin* ^*WT*^ *-EGFP*

*dnlg2* ^*KO70*^ *; C57-GAL4 > UAS-Cofilin* ^*S3A*^ *-EGFP*

*dnlg2* ^*KO70*^ *; C57-GAL4 > UAS-Cofilin* ^*S3E*^ *-EGFP*

We generated *UAS-Cofilin-EGFP* by inserting the full-length Cofilin protein coding sequence into pUAST-EGFP-attB vector, then Xia Yao (Qing Zhang lab, Model Animal Research Center of Nanjing University) helped to injected the resulting plasmids into embryos of attP transgenic flies (BDSC, 35568). Transgenic fly strains were generated based on the φ31-mediated integration system using the landing site at the cytological position 87B^73^. The transgenes were subsequently crossed into a *w*^*1118*^ background. For *UAS-Cofilin*^*S3A*^*-EGFP* and *UAS-Cofilin*^*S3E*^*-EGFP* strains, we changed two or three bases so the Serine3 at the phosphorylation site can be changed to Alanine or Glutamic residue.

cDNA PCR with the following primers were performed to generate the recombinant plasmids.

cofilin-F: 5’-tgaatagggaattgggaattcATGGCTTCTGGTGTAACTGTGTCTG-3’;

cofilinS3A-F: 5’-tgaatagggaattgggaattcATGGCTGCGGGTGTAACTGTGTCTG-3’;

cofilinS3E-F: 5’-tgaatagggaattgggaattcATGGCTGAGGGTGTAACTGTGTCTG-3’;

cofilin-R: 5’-gatctgcgcgttaacgaattcTTATTGGCGGTCGGTGGC-3’.

These transgenic strains were verified by PCR and Immunochemistry analysis:

verify-F: 5’-GTAACCAGCAACCAAGTAAATC-3’;

verify-R: 5’-TAAATCTCTGTAGGTAGTTTGTCC-3’.

### Mice

C57BL/6 mice were used as wildtype mice, purchased from Huangchuang Sino. Nlg1^KO^ mice were generated previously^74^. The mice were housed under standard laboratory conditions with access to food and water ad libitum, stable temperature (22 ± 1 °C), and 12-h light–dark cycle (lights on at 07:00). All animal care and experimental procedures were followed by the Animal Experimental Ethical Guide of Southeast University and Animal Core Facility of Nanjing Medical University.

The striatum tissues from 3-4 week-old mice (two males and two females) were used for the western blot experiments detecting the RACK1 contents. The cortex and hippocampus tissues from 3-4 week-old male C57BL/6 mice were used for the co-IP experiments.

### Larval locomotion activity detection

To monitor larval locomotion, transparent dishes (diameter, 8.5 cm) with 3.5% agar substrate were used as an arena for crawling larvae. Grape purple food colorant was added until the substrate presented a dark purple color. In each trial, a single wandering third-instar larvae was transported to the center of the arena. The movement of larvae was visualized via a standard commercial video camera (resolution, 640 × 480) for 3 min. Tracker software was written in Python to track the trajectory, and 3-min trajectory distances were calculated by AIM-LSM for assessing larval locomotion activity.

### Western blot analysis

In brief, adult fruit fly heads or third-instar larvae body-wall muscle were homogenized with 2 × SDS loading buffer (Takara, H621), and the total protein lysates were separated by precast protein gel (GenScript, Sure PAGE, M00653) and electro-transferred onto polyvinylidene difluoride membranes. Immobilized proteins on the membrane were probed with proper primary antibodies at shaker in 4 °C overnight. The samples were then incubated with HRP-conjugated secondary antibodies at room temperature for 2 h. The targeted proteins were visualized with high-sig ECL western blotting substrate from Tanon and SuperSignal West Femto Maximum Sensitivity substrate from Thermo Scientific. The blots shown are representative of ≥3 independent experiments. The antibodies used in this study can be found in Supplementary Data. 1.

### Immunoprecipitation

Briefly, adult heads were homogenized and lysed in ice-cold cell lysis buffer for Western and IP (Beyotime, P0013J) added EDTA-free Protease Inhibitor Cocktail (Roche). The lysates were kept on shaker at 4 °C for 60 min and centrifuged at 12,000 rpm at 4 °C for 15 min, twice. The supernatant was incubated with proper antibody on shaker at 4 °C overnight, and incubated with Pierce Protein A/G Agarose (Thermo Scientific) at room temperature for 60 min in the second day. Then the lysates were centrifuged at 2000 rpm at 4 °C for 2 min and discard the supernatant. The Agarose was subsequently washed three times with lysis buffer, and proteins were eluted by boiling the beads in 2 × SDS loading buffer. Following, centrifuged at 12,000 rpm at room temperature for 2 min and the supernatant was used for western blot analysis.

For in vitro immunoprecipitation, N-terminal (extracellular part containing the esterase-like domain, residues Q37-Y684) and C-terminal (transmembrane and intracellular domains, residues L685-V1248) fragments of DNlg2 were synthesized bound to the Strep tag II, respectively. GFP-Strep tag II was used as the negative control. Proteins were enriched by MagStrep beads (iba, 2-4090-002). Input and bound fractions were analyzed by immunoblotting. The antibodies used in this study can be found in Supplementary Data. 1.

### NMJ staining and image analysis

In brief, the body-wall muscles from third instar larvae were dissected in PBS solution and fixed them for 40 min with 4% paraformaldehyde or for 5 min with chilled methanol. Then, the fixed samples were washed four times in 0.3% PBST (0.3% Triton X-100 in PBS), blocked in blocking solution (0.5% BSA in 0.3% PBST) for 1 h, and incubated with primary antibody at 4 °C overnight. Secondary antibodies were incubated at room temperature for 2 h. Then the samples were washed extensively and mounted in VectaShield mounting medium (Vector Laboratories). All images were collected using an LSM 710 Confocal Station (Zeiss) and analyzed by Image J software. The antibodies used in this study can be found in Supplementary Data. 1.

For measurements of fluorescence intensity, we set an arbitrary threshold for each channel based on the difference in intensity between the NMJ and the background regions. The sum of the pixels with intensities above the threshold was recorded by ImageJ. For comparison of fluorescence intensities between genotypes, all samples were processed simultaneously and under identical conditions. Anti-HRP staining signal was used as a control signal.

For F-actin staining, NMJ samples were incubated with a high concentration of Texas Red-conjugated phalloidin (1:6) for 13 min at room temperature after secondary antibodies incubation and cleaning. Images were collected at muscles 12/13, because the distance between the boutons and the myofibril in that muscle is relatively large, thus reducing the interference from a strong F-actin-stained background due to myofibril^75^. We performed statistical analyses as described in the source data tables.

Quantification of active zones was performed using a previous modified method^7^. The apposed active zones were defined as active zones correspond to GluRs. We co-applied GluRIIB rabbit polyclonal antibody and BRP mouse monoclonal antibody (nc82) for immunofluorescence staining. A branch of bouton clusters (usually 8–10 boutons) from muscle 4 of segment A3 were included in the analysis instead of quantifying only terminal boutons. To avoid subjective variation in the counting the numbers of non-overlapping BRP/GluRIIB spots, the ratio of overlapping BRP area to the total BRP area, namely percentage apposed AZ area was used to represent the extent of apposition. That method is very similar to the way that the extent of co-localization of presynaptic and postsynaptic proteins in mammalian systems is quantified^76^. The BRP spots in single-channel images and the overlapping spots of BRP and GluRIIB in corresponding two-channel merged images were respectively detected using ImageJ. Then, the total area of the BRP spots and that of the BRP/GluRIIB overlapping spots were quantified using the ‘analyze particles’ function in ImageJ. We defined the relative apposed active zone as the ratio of the total BRP/GluRIIB overlapping spot area to the total BRP spot area.

### Rac1 activity assay

The heads of approximately 500 adult flies or the body wall muscle of approximately 300 third instar larvae were homogenized and lysed to gain sample. For detecting the relative levels of active Rac1, Pak-PBD pull-down assay (Thermo Scientific, 16118) was used. The anti-Rac1 monoclonal antibody (1:1000 dilution) was used to detect total and active Rac1 levels. The Rac1-GTP relative amount is calculated by dividing Rac1-GTP by tubulin.

### Rho activity assay

The heads of approximately 500 adult flies were homogenized and lysed to gain sample. For detecting the relative levels of active Rho, Rhotekin-RBD pull-down assay (Thermo Scientific, 16116) was used. The anti-Rho monoclonal antibody (1:1000 dilution) was used to detect total and active Rho levels. The Rho-GTP relative amount is calculated by dividing Rho-GTP by tubulin.

### Electrophysiology

Third instar larvae were dissected and intracellular membrane potentials were recorded as previously described^14^. Briefly, wandering third instar larvae were dissected in Ca^2+^-free HL3.1 saline, fat and gut in the body were removed, brain and VNC were cut, and the body wall was carefully spread out to expose muscle for avoiding damage the muscle architecture. Then HL3.1 saline was used to wash and immerser the sample. We chose muscle 6 in the A3 segment for recording using the recording electrodes (20–50 MΩ) filled with 3 M KCl. Miniature EJPs (mEJPs) were recorded for a period of 60 s. All recordings were conducted at room temperature with an Axoclamp 900 A amplifier (Molecular Devices, Sunnyvale, CA) in bridge mode. The data were digitized with a Digitizer 1322 A (Molecular Devices). We used Clampfit 10.2 to analyze the data. Only the recordings with resting membrane potentials ranging from −60 to −65 mV were used for analysis.

All recordings were conducted in modified HL3.1 solution containing 70 mM NaCl, 5 mM KCl, 4 mM MgCl_2_, 10 mM NaHCO_3_, 0.6 mM CaCl_2_, 115 mM sucrose, 5 mM trehalose and 5 mM HEPES, pH 7.2.

### Mass spectrometry

To enrich DNlg2 and its interacting proteins, DNlg2 tagged with EGFP was overexpressed using a ubiquitous driver (*da-GAL4*), and the protein complexes were extracted from lysates of *Drosophila* adult head tissue using the GFP-Trap Agarose (Chromotek). GFP overexpression served as the negative control. Afterwards, the fresh protein lysates were sent to Shanghai Applied Protein Technology Company for LC-MS. Trypsin was utilized as the incision enzyme. Proteins for *Drosophila melanogaster* from UniProt were used as a database and for analysis.

For the MS to detect the interacting proteins of the two terminals of DNlg2, the GST-tagged extracellular (M1-S965) and intracellular (A989-V1248) fragments of DNlg2 were expressed in the *E. Coli* and purified, respectively. These two fragments were then incubated with the fresh lysates of wildtype *Drosophila* adult head tissue, and further pulled down the protein complexes using the GST antibody and Pierce Protein A/G Agarose (Thermo Scientific).

### Statistics and Reproducibility

All results are presented as mean ± SEM, and the data were analyzed using GraphPad Prism 9.0 software and Microsoft Excel. We assessed the significance between two groups using the two-tailed Student’s *t* test, or the ordinary One-way ANOVA with multiple comparisons. A value of *P* < 0.05 was considered statistically significant. Asterisks indicate significant differences between genotypes: *p < 0.05, **p < 0.01, ***p < 0.001, ****p < 0.0001.

### Reporting summary

Further information on research design is available in the Nature Portfolio Reporting Summary linked to this article.

### Supplementary information

supplementary Figure 1 and 2

Description of Additional Supplementary Data

Supplementary data 1

Supplementary data 2

Reporting Summary

## Data Availability

All data supporting the findings of this study are included in the article and its Supplementary Information. Key resources, p-values and numerical source data for all graphs can be found in Supplementary Data. 1, while the original uncropped western blot images can be found in Supplementary Data. 2. The MS proteomics data have been deposited to the ProteomeXchange Consortium via the PRIDE^77^ partner repository (https://www.ebi.ac.uk/pride/archive/projects/PXD045754).
