# Supplementary material for: Neuroligin 2 governs synaptic morphology and function through RACK1-cofilin signaling in Drosophila
Source: Commun Biol. 2023 Oct 18;6:1056. doi: 10.1038/s42003-023-05428-3 (PMC10584876; doi:10.1038/s42003-023-05428-3)
Supplement: Supplementary file 1 — supplementary Figure 1 and 2 [file 42003_2023_5428_MOESM1_ESM.pdf]

a

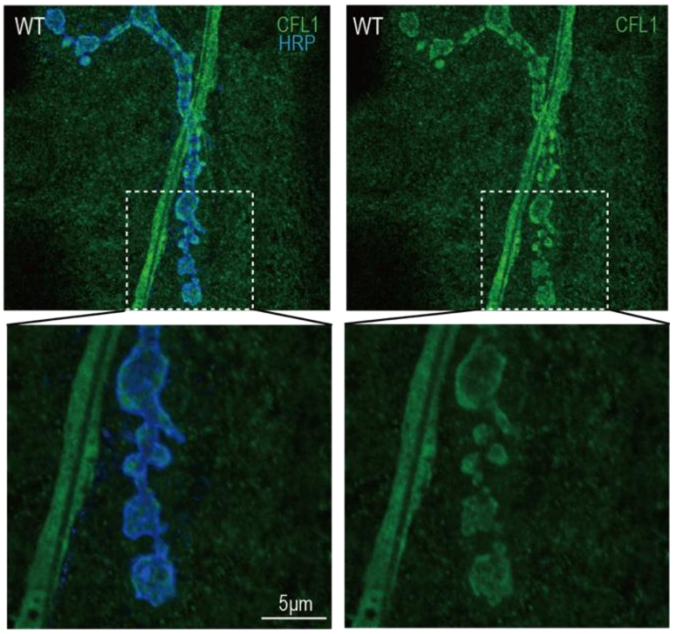

Supplementary Fig. 1 Localization of total Cofilin at WT NMJ.

a Confocal images of type Ib boutons at WT third instar larvae NMJ muscle 4 labeled with anti-CF1 (green, Cofilin) and anti-HRP (blue).

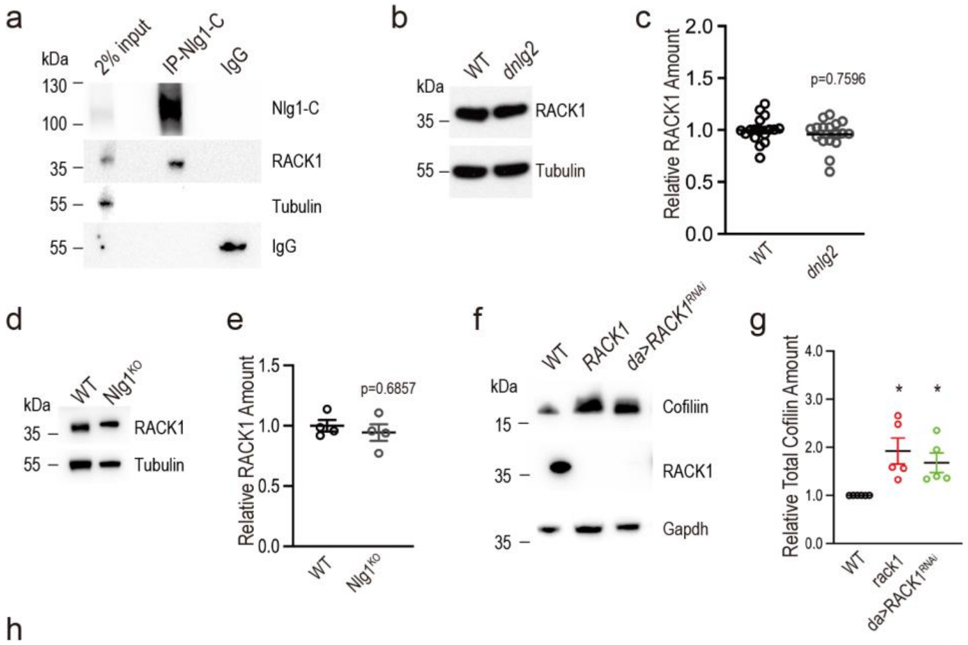

h

Protein	Species	% Identity	% Similarity (Blossom 45 with threshold 0)
RACK1	*Homo sapiens*	76.656	91.798
	Drosophila melanogaster		
Rac1	*Homo sapiens*	91.667	96.875
	Drosophila melanogaster		

i

Species	Protein	Delta G (binding free energy)	Kd (dissociation constant)
Drosophila melanogaster	Rac1	-9.75 kcal/mol	7.11e-08 M
	RACK1		
Homo sapiens	Rac1	-10.38 kcal/mol	2.44e-08 M
	RACK1		
Mus musculus	Rac1	-11.75 kcal/mol	2.42e-09 M
	RACK1		

Supplementary Fig. 2 The interaction of Neuroligin-RACK1-Rac1 is conserved in mice.

a Neuroligin1 can interact with RACK1 in mice. IP from brain tissue of wildtype mouse with anti-Neuroligin1 antibody.

b Western blots show the protein level of RACK1 in WT (n=17) and *dnlg2* mutants (n=17) third instar larvae muscle.

c Scatter diagram of the RACK1 relative amount in (b). There was no change in the RACK1 expression in *dnlg2* mutants.

d Western blots show the protein level of RACK1 of brain tissue from WT (n=4) and $Nlg1^{KO}$ mice (n=4).

e Scatter diagram of the RACK1 relative amount in (d). There was no change in the RACK1 expression in $Nlg1^{KO}$ mice.

f Western blots show the protein level of RACK1 in WT (n=6), *RACK1* mutants (n=5), and *da>RACK1*^{RNAi} (n=5) third instar larvae muscle.

g Scatter diagram of the RACK1 relative amount in (f). The RACK1 expression were increased in *RACK1* mutants and *da>RACK1*^{RNAi} third instar larvae muscle

h Homology analysis of RACK1 and Rac1 in *Homo sapiens* and *Drosophila melanogaster*. In general, identify more that 30%, similarity more than 50% can speculate that these two proteins are homologous.

i Binding free energy and dissociation constant between Rac1 and RACK1 in *Drosophila melanogaster*, *Homo sapiens* and *Mus musculus*. The lower the Delta G and Kd are, the more likely these two proteins can bind with each other. This calculation was run on Protein-Protein Affinity Predictors (https://www.iitm.ac.in/bioinfo/PPA_Pred/prediction.html).
