# Supplementary material for: Neuroligin 2 governs synaptic morphology and function through RACK1-cofilin signaling in Drosophila
Source: Commun Biol. 2023 Oct 18;6:1056. doi: 10.1038/s42003-023-05428-3 (PMC10584876; doi:10.1038/s42003-023-05428-3)
Supplement: Supplementary file 2 — Description of Additional Supplementary Data [file 42003_2023_5428_MOESM2_ESM.docx]

**Description of Additional Supplementary Files**

**File name:** Supplementary Data 1

**Description:** key resources+p values+ numerical source data 2.

**File name:** Supplementary Data 2

**Description:** WB source pics
