# Supplementary material for: Neuroligin 2 governs synaptic morphology and function through RACK1-cofilin signaling in Drosophila
Source: Commun Biol. 2023 Oct 18;6:1056. doi: 10.1038/s42003-023-05428-3 (PMC10584876; doi:10.1038/s42003-023-05428-3)
Supplement: Supplementary file 3 — Supplementary data 1 [file 42003_2023_5428_MOESM3_ESM.pdf]

Reagent type (species) or resource	Designation	Source or reference	Identifiers	Additional information
genetic reagent (D. *melanogaster*)	dnlg1$^{ex1.9}$/TM6B	PMID: 20547130		
genetic reagent (D. *melanogaster*)	dnlg1$^{ex2.3}$/TM6B	PMID: 20547130		
genetic reagent (D. *melanogaster*)	dnlg2^{KO70}	PMID: 21228178		
genetic reagent (D. *melanogaster*)	tsr^{N96A}/Cyo	Bloomington Drosophila Stock Center	9108	
genetic reagent (D. *melanogaster*)	C57-Gal4	PMID: 8893021		V. Budnik (University of Mas-sachusetts School of Medicine, Worcester, MA)
genetic reagent (D. *melanogaster*)	da-Gal4	Bloomington Drosophila Stock Center	55851	daughterless-GAL4
genetic reagent (D. *melanogaster*)	UAS-Cofilin-EGFP	lab generated; this paper		
genetic reagent (D. *melanogaster*)	UAS-Cofilin^{S3A}-EGFP	lab generated; this paper		
genetic reagent (D. *melanogaster*)	UAS-Cofilin^{S3E}-EGFP	lab generated; this paper		
genetic reagent (D. *melanogaster*)	UAS-DNlg2-HA	lab generated		
genetic reagent (D. *melanogaster*)	Rack1^{EY00128}	Bloomington Drosophila Stock Center	15000	
genetic reagent (D. *melanogaster*)	Rack1$^{1.8}$	Bloomington Drosophila Stock Center	24152	
genetic reagent (D. *melanogaster*)	Rack1^{RNAi}	VDRC	V27858	
genetic reagent (D. *melanogaster*)	UAS-Myr-RACK1-HA	gift from Zizhang Zhou (Shandong Agricultural University, China)		

genetic reagent (M. musculus)	C57BL/6	Huangchuang Sino		
genetic reagent (M. musculus)	$Nlg1^{KO}$	lab generated		
cell line (Homo sapiens)	HEK 293T	China Center for Type Culture Collection	(RRID:CVCL_0063)	
antibody	anti-DLG	Developmental Studies Hybridoma Bank	DSHB:4F3; RRID: AB_528203	1:100 for IHC
antibody	anti-BRP	Developmental Studies Hybridoma Bank	DSHB:NC82; RRID:AB_2314866	1:25 for IHC
antibody	anti-GluRIIA	Developmental Studies Hybridoma Bank	DSHB:8B4D2; RRID:AB_528269	1:25 for IHC
antibody	anti-GluRIIB	gift from Yongqing Zhang, Chinese Academy of Sciences		1:2500 for IHC
antibody	anti-DPak	gift from Nicholas Harden, Simon Fraser University, Burnaby, BC, Canada		1:200 for IHC
antibody	anti-DNase I (G-actin)	Invitrogen	Invitrogen: PA5-76783; RRID: AB_2720510	1:100 for IHC
antibody	anti-HRP	Jackson ImmunoResearch	Jackson ImmunoResearch:323-005-021 RRID: AB_2314648	1:1000 for IHC
antibody	Alexa 488-, 555-, or 405 -secondaries	Invitrogen		1:500 for IHC
Dye	Texas Red-conjugated Phalloidin	Molecular Probes	ThermoFisher: T7471	1:10 for IHC
antibody	anti-p-Cofilin	Santa Cruz	Santa Cruz: 12912(discontinued)	1:700 for WB
antibody	anti-Cofilin	gift from James R. Bamburg, Colorado State University		1:10000 for WB
antibody	anti-twinstar	gift from Tadashi Uemura, Kyoto University		1:2000 for WB

antibody	anti-RACK1	Cell Signaling Technology	CST: 5432S	1:1000 for WB
antibody	anti-Neuroligin1	Synaptic System	Synaptic System: 129013	1:100 for IP, 1:1000 for WB
antibody	anti-DNlg2	lab generated		1:200 for WB
antibody	anti-HA	Sigma	Sigma: H9658	1:200 for IP, 1:2000 for WB
antibody	anti-GFP(N-terminal)	Sigma	Sigma: G1544	1:100 for IP, 1:1000 for WB
antibody	anti mcherry	Abcam	Abcam	1:1000 for WB
antibody	anti-Strep II-Tag	ABconal	Abconal: AE066	1:1001 for WB
antibody	Rabbit Control IgG	ABconal	Abconal: AC005; RRID: AB_2771930	1:100 for IP
antibody	Mouse Control IgG	ABconal	Abconal: AC011; RRID: AB_2770414	1:100 for IP
antibody	anti-Tubulin	Sigma	Sigma: Clone DM1A; RRID: AB_477593	1:10000 for WB
antibody	Mouse Anti-Gapdh	Abcam	Abcam: ab8245	1:1000 for WB
antibody	Mouse anti-Synataxin	Developmental Studies Hybridoma Bank	DSHB:8C3; RRID: AB_528484	1:1000 for WB
antibody	Goat anti-rabbit IgG(H+L) Secondary Antibody, HRP	Invitrogen	Invitrogen: 31460; RRID: AB_228341	1:10000 for WB
antibody	Goat anti-mouse IgG(H+L) Secondary Antibody, HRP	Invitrogen	Invitrogen: 31430; RRID: AB_228307	1:10000 for WB
antibody	Goat Anti-Mouse IgG LCS Secondary Antibody, HRP	Abbkine	Abbkine: A25012	1:10000 for WB
antibody	Goat Anti-Mouse IgG HCS Secondary Antibody, HRP	Abbkine	Abbkine: A25112	1:10000 for WB
antibody	Mouse Anti-Rabbit IgG LCS Secondary Antibody, HRP	Abbkine	Abbkine: A25022	1:10000 for WB
antibody	Goat Anti-Rabbit IgG HCS Secondary Antibody, HRP	Abbkine	Abbkine: A25222	1:10000 for WB

Assay Kit	Active Rac1 Pull-Down and Detection Kit	Thermo Scientific	Thermo Scientific: 16118
Assay Kit	Active Rho Pull-Down and Detection Kit	Thermo Scientific	Thermo Scientific: 16116
other	Pierce™ Protein A/G Agarose	Thermo Scientific	Thermo Scientific: 20421
other	GFP-Trap Agarose	Chromotek	
other	MagStrep type 3 bead	iba	iba: 2-4090-002

Figure	genotypes	p-value	summary
Fig.1b	WT VS *dnlg2*	0.0324	*
	WT VS *cofilin*	0.0488	*
	dnlg2 VS *cofilin*	0.9926	ns
Fig.1d	WT VS *dnlg1*	0.0029	**
	WT VS *dnlg2*	0.0003	***
	WT VS *cofilin*	0.0182	*
	dnlg1 VS *dnlg2*	0.9763	ns
	dnlg2 VS *cofilin*	>0.9999	ns
Fig.1f	WT VS *dnlg1*	0.0048	**
	WT VS *dnlg2*	0.001	**
	WT VS *cofilin*	0.0047	**
	dnlg1 VS *dnlg2*	0.9421	ns
	dnlg1 VS *cofilin*	0.9963	ns
	dnlg2 VS cofilin	0.9891	ns
Fig.2b	WT VS *dnlg1*	0.0063	**
	WT VS *dnlg2*	0.0489	*
	dnlg1 VS *dnlg2*	0.7714	ns
Fig.2d	WT VS *dnlg1*	0.1878	ns
	WT VS *dnlg2*	>0.9999	ns
	dnlg1 VS *dnlg2*	0.4974	ns
Fig.2f	WT VS *dnlg2*	<0.0001	****
	WT VS *dnlg2; C57>*$Cofilin^{WT}$	0.0043	**
	WT VS *dnlg2; C57>*$Cofilin^{S3A}$	0.2604	ns
	WT VS *dnlg2; C57>*$Cofilin^{S3E}$	>0.9999	ns

	dnlg2 VS *dnlg2; C57>Cofilin*^{WT}	>0.9999	ns
	dnlg2 VS *dnlg2; C57>Cofilin*^{S3A}	0.0487	*
	dnlg2 VS *dnlg2; C57>Cofilin*^{S3E}	<0.0001	****
Fig.2h	WT VS *dnlg2*	0.0003	***
	WT VS *dnlg2; C57>Cofilin*^{WT}	0.1428	ns
	WT VS *dnlg2; C57>Cofilin*^{S3A}	0.5628	ns
	WT VS *dnlg2; C57>Cofilin*^{S3E}	0.0019	**
	dnlg2 VS *dnlg2; C57>Cofilin*^{WT}	0.2289	ns
	dnlg2 VS *dnlg2; C57>Cofilin*^{S3A}	0.0449	*
	dnlg2 VS *dnlg2; C57>Cofilin*^{S3E}	0.9745	ns
Fig.3b	WT VS *dnlg2*	<0.0001	****
	WT VS *dnlg2; C57>Cofilin*^{WT}	0.3054	ns
	WT VS *dnlg2; C57>Cofilin*^{S3A}	0.9979	ns
	WT VS *dnlg2; C57>Cofilin*^{S3E}	0.0149	*
	dnlg2 VS *dnlg2; C57>Cofilin*^{WT}	0.0432	*
	dnlg2 VS *dnlg2; C57>Cofilin*^{S3A}	0.0002	***
	dnlg2 VS *dnlg2; C57>Cofilin*^{S3E}	<0.0001	****
Fig.3d	WT VS *dnlg2*	<0.0001	****
	WT VS *dnlg2; C57>Cofilin*^{WT}	0.9733	ns
	WT VS *dnlg2; C57>Cofilin*^{S3A}	>0.9999	ns
	WT VS *dnlg2; C57>Cofilin*^{S3E}	0.0002	***
	dnlg2 VS *dnlg2; C57>Cofilin*^{WT}	<0.0001	****
	dnlg2 VS *dnlg2; C57>Cofilin*^{S3A}	<0.0001	****
	dnlg2 VS *dnlg2; C57>Cofilin*^{S3E}	0.215	ns
Fig.4b	WT VS *dnlg2*	0.0049	**

	WT VS *dnlg2; C57>Cofilin*^{WT}	0.0652	ns
	WT VS *dnlg2; C57>Cofilin*^{S3A}	0.1273	ns
	WT VS *dnlg2; C57>Cofilin*^{S3E}	0.0188	*
	dnlg2 VS *dnlg2; C57>Cofilin*^{WT}	<0.0001	****
	dnlg2 VS *dnlg2; C57>Cofilin*^{S3A}	<0.0001	****
	dnlg2 VS *dnlg2; C57>Cofilin*^{S3E}	0.9979	ns
Fig.4c	WT VS *dnlg2*	0.0009	***
	WT VS *dnlg2; C57>Cofilin*^{WT}	0.139	ns
	WT VS *dnlg2; C57>Cofilin*^{S3A}	>0.9999	ns
	WT VS *dnlg2; C57>Cofilin*^{S3E}	0.4923	ns
	dnlg2 VS *dnlg2; C57>Cofilin*^{WT}	<0.0001	****
	dnlg2 VS *dnlg2; C57>Cofilin*^{S3A}	0.0013	**
	dnlg2 VS *dnlg2; C57>Cofilin*^{S3E}	0.2372	ns
Fig.4e	WT VS *dnlg2*	<0.0001	****
	WT VS *dnlg2; C57>Cofilin*^{WT}	0.029	*
	WT VS *dnlg2; C57>Cofilin*^{S3A}	0.6554	ns
	WT VS *dnlg2; C57>Cofilin*^{S3E}	<0.0001	****
	dnlg2 VS *dnlg2; C57>Cofilin*^{WT}	<0.0001	****
	dnlg2 VS *dnlg2; C57>Cofilin*^{S3A}	<0.0001	****
	dnlg2 VS *dnlg2; C57>Cofilin*^{S3E}	0.9972	ns
Fig.5f	WT VS *dnlg2*	0.0079	**
Fig.5h	WT VS *dnlg2*	0.0079	**
Fig.5l	*C57>RACK1* VS *dnlg2;C57>RACK1*	<0.0001	****

Fig.5n	WT VS *da>RACK1*^{RNAi}	0.0286	*
Supp Fig.2c	WT VS *dnlg2*	0.7596	ns
Supp Fig.2e	WT VS Nlg1^{KO}	0.6857	ns
Supp Fig.2g	WT VS *rack1*	0.0143	*
	WT VS *da>RACK1*^{RNAi}	0.0452	*

Fig.1b	WT	Dnlg2	Cofilin
	1.744363	1.692803	1.107304
	1.174786	1.060086	1.445103
	1.855502	0.827371	1.487059
	0.70142	1.467468	1.470437
	0.951797	1.075643	1.151485
	0.744313	0.591209	0.894411
	3.130543	1.470768	0.87853
	0.864239	1.392138	1.327127
	1.01228	1.50223	1.519275
	1.186036	1.303574	0.809759
	2.214342	0.843378	1.240574
	2.018549	0.956599	1.047554
	1.890574	1.12793	1.186622
	2.571137	1.337023	1.205783
	1.40141	1.019086	
	1.435055		
	1.95784		
	2.124795		
	1.368097		

Fig.1d	WT	Dnlg1	Dnlg2	Cofilin
	0.603509	0.832096	0.930419	0.721964
	0.451943	0.856312	1.07072	0.862763
	0.407221	1.01562	1.226716	0.678852
	0.341139	0.905908	0.928074	0.985355
	0.641573	0.862413	1.167241	1.037998
	0.645733	0.72454	1.113469	1.172356
	0.478609	1.057631	1.188182	1.323974
	1.052555	0.581738	0.533445	1.132162
	0.78031	0.629577	0.968054	1.259323

	0.597264	0.762214	0.946862	
	0.650663	0.692662	0.801996	
	0.783329	0.761815	0.768232	
	0.830104	0.757668	0.788331	
	0.475677	0.907924	0.806762	
	0.613021	1.165316	1.767847	
	0.669041	1.479355	0.935859	
	0.834823	1.733281	0.820812	
	0.810664	1.480547	0.781716	
	0.969525	1.120517	1.180748	
	0.911045	1.131625	0.94243	
	0.669319	1.156552	1.95199	
	0.728662	1.371616	1.323813	
	0.694623	0.634836	1.187607	
	0.724136		0.927093	
	0.717715		0.744823	
	0.900862		0.771817	
	0.914971		1.003183	
	0.858989		0.88275	
	1.133464		0.962766	
			0.978064	

Fig.1f	WT	*dnlg1*	*dnlg2*	*cofilin*
	1.238507	0.803317	0.747996	0.325291
	1.10728	0.583606	0.698439	1.007953
	1.067145	0.973551	0.423755	0.69826
	0.995859	0.643975	0.708368	0.913343
	1.413036	0.834038	0.674578	0.549907
	0.508836	0.483066	0.478982	0.690427
	0.925645	0.694174	0.781044	0.533306
	0.985996	0.458652	0.8296	0.619487

	0.872935	0.763882	0.495525
	0.884755	0.632756	0.547402

Fig.2b	WT	Dnlg1	Dnlg2
	1	0.101911	0.615303
	1	0.444412	0.870877
	1	0.597356	0.579855
	1	0.878184	0.594798
	1		

Fig.2d	WT	Dnlg1	Dnlg2
	0.962582	1.062051	1.057804
	1.010559	1.034941	1.050426
	1.026859	1.088971	1.010324
	0.965594	1.303577	0.836815
	0.915101	0.98583	1.030656
	1.141149	1.127966	1.259641
	0.978157	1.302701	1.030178
	1.368426	0.78127	0.915646
	0.869907	0.979645	0.830551
	0.873963		0.876056
	0.887705		0.919168
	1.040852		1.008932
	0.896414		0.939125
	1.084375		0.928962
	0.978359		1.412092
	0.980575		1.017131
	1.019425		1.168426
	1.280015		1.160897
	1.014652		0.974322

	0.927	1.463912
	0.802892	0.546606
	0.655025	0.840711
	0.889317	0.963664

Fig.2f	WT	*dnlg2*	C57>cofilin;*dnlg2*	C57>cofilin^{S3A};*dnlg2*	C57>cofilin-^{S3E};*dnlg2*
	1.5272	0.836634	2.094841	1.913624	1.541488
	1.290249	0.838947	1.230995	1.491682	2.198437
	1.410519	0.909797	0.9440664	1.54833	1.929725
	1.726045	0.632428	1.074027	0.9411108	1.339441
	1.541575	0.80218	0.8453785	0.9610088	2.297982
	2.131573	1.116031	1.147163	1.17734	2.639916
	1.374852	1.228669	1.103408	1.414271	1.703957
	2.051897	1.13438	1.006815	1.902869	1.604532
	1.769817	0.948389	1.053017	1.46839	1.726827
	2.13521	1.38499	1.470236	0.990692	1.617319
	1.817666	0.831515	0.8250654	1.121896	2.42779
	2.172481	1.158928	1.43373	1.282012	1.196854
		0.855495	1.312436	1.170132	1.299384
		1.390979	1.212457	1.415285	
		1.359513	1.108529	1.345767	
			1.100441	1.127481	
			1.840701	1.298599	
				1.413445	
				1.431319	

Fig.2h	WT	*dnlg2*	C57>cofilin;dnl*g2*	C57>cofilin^{S3A};dnl*g2*	C57>cofilin^{S3E};dnl*g2*
	115	81	73	111	105
	81	67	67	120	70
	82	54	45	71	75

	84	72	105	99	63
	84	67	78	98	114
	125	48	111	97	77
	91	89	99	58	49
	95	88	68	72	43
	88	67	74	77	50
	113	54	108	83	81
	121	46	87	69	58
	94	63	54		56
	105	49			61

Fig.3b	WT	*dnlg2*	*dnlg2; C57>Cofilin*^{WT}	*dnlg2; C57>Cofilin*^{S3A}	*dnlg2; C57>Cofilin*^{S3E}
	0.439259	0.479619	0.5748774	0.6132042	0.5800968
	0.674916	0.415155	0.5725594	0.5091501	1.021045
	0.608385	0.444283	0.502178	0.8829735	0.5671043
	0.676214	0.46499	0.4823358	0.5745794	0.8419036
	0.642254	0.448131	0.474611	0.6350752	0.8056206
	0.428941	0.416269	0.4548775	0.6303945	0.8448813
	0.547163	0.476524	0.4367465	0.5129529	0.6579048
	0.525063	0.397869	0.4723042	0.6082621	0.7871047
	0.696593	0.356769	0.4483627	0.5831423	0.7131712
	0.768968	0.375677	0.5246949	0.5133775	0.5850168
	0.507234	0.366473	0.6855046	0.5243551	0.765036
	0.727568	0.382554	0.75878	0.5349102	0.6135246
	0.525312	0.406187		0.6539939	0.8879363
	0.725246	0.400118			0.6057319
	0.607075	0.411291			0.5045575
	0.666285	0.435752			0.9226329

Fig.3d	WT	*dnlg2*	C57>cofilin;dnlg2^{70}	C57>cofilin^{S3A};dnlg2	C57>cofilin^{S3E};dnlg2

	0	11.11111	0	0	6.25
	0	18.18182	0	0	11.11111
	0	11.11111	0	0	0
	0	14.28571	0	0	5.882353
	0	9.090909	0	0	9.090909
	0	8.333334	7.142857	0	4.761905
	0	7.692308	0	0	15.78947
	0	7.142857	0	0	

Fig.4b	WT	*dnlg2*	C57>cofilin;*dnlg2*	C57>cofilin^{S3A};*dnlg2*	C57>cofilin^{S3E};*dnlg2*
	0.601818	0.547438	1.096243	0.8418113	0.6998644
	0.587226	0.511136	0.8342898	0.6969539	0.3478413
	0.601992	0.392806	0.7151055	0.7428488	0.5587777
	0.676477	0.396173	0.5533867	0.6985663	0.4153913
	0.665038	0.401462	0.8230125	0.6801827	0.6337013
	0.739873	0.527524	0.6739306	0.8939684	0.6466234
	0.626461	0.39049	0.6105897	0.895314	0.4639844
	0.475268	0.351217	0.5623466	0.6581864	0.424096
	0.500952	0.414155	0.6241474	0.6688919	0.7154874
	0.632702	0.705021	0.8932857	0.7012316	0.5593274
	0.604861	0.481178	0.9121483	0.5597317	0.4741386
	0.4425	0.616317	0.8410707	0.7055294	0.515066
	0.727731	0.532619	0.6403174	0.676713	0.4925
	0.630516	0.425844		0.7258494	0.6687047
	0.566263	0.697821		0.7305976	0.5593987
	0.455798	0.588662		0.7378117	0.3865797
	0.783498	0.587311		0.6620972	0.3474608
	0.606523	0.528768		0.8211702	
	0.495103	0.626042		0.7666907	
	0.757615			0.6611214	
	0.922701			0.6490787	

	0.641364	0.8089136	
	0.777435		
	0.902024		

Fig.4c	WT	*dnlg2*	C57>cofilin;dnlg2	C57>cofilin^{S3A};dnlg2	C57>cofilin^{S3E};dnlg2
	2.1	0.8	2.333333	1.766667	1.966667
	1.916667	0.983333	3.45	3.616667	1.283333
	2.1	0.8	3	2.866667	0.7666667
	1.916667	0.983333	2	1.383333	0.7666667
	2.15	1.033333	3.016667	1.733333	1.283333
	1.783333	0.866667	2.666667	3.166667	2.566667
	1.766667	0.433333	2.4	1.433333	1.066667
	1.7	0.35	1.95	1.966667	2.083333
	0.933333	0.816667	1.25	1.233333	1.983333
	1.05	1.15	2.033333	1.583333	1.883333
	2.066667	0.566667	1.166667	1.366667	1.683333
	2.033333	1.4	3.933333	1.133333	1.766667
	1.766667	1.616667	3.3	1.916667	0.9666666
	1.3	1.216667	2.783333	1.55	2.483333
	1.033333	0.683333		1.366667	2.466667
	2.85	2.1		2.566667	1.15
	1.816667	1.816667		2.4	1.7
	3.283333	2.233333		4.016667	
	1.85	1.583333		1.616667	
	1.933333	1.083333		2.333333	
	1.816667	1.5		1.483333	
	3.116667	1.583333		1.35	
	0.916667	1.583333			
	3.216667				
	3.55				
	1.65				

Fig.4e	WT	*dnlg2*	C57>cofilin;*dnlg2*	C57>cofilin^{S3A};*dnlg2*	C57>cofilin^{S3E};*dnlg2*
	175.2489	122.8103	105.9897	131.3896	111.0402
	115.6703	32.21412	40.87357	89.00614	67.91529
	131.5253	41.88961	31.31561	80.11279	52.74792
	156.8545	53.68615	74.00159	90.78528	43.16989
	92.87393	40.40607	36.83167	90.83129	114.444
	94.32861	84.60754	79.73235	79.80708	46.43389
	111.2574	38.41648	95.00436	120.5227	54.48031
	102.5711	60.16329	60.38561	88.94283	30.12854
	157.2553	52.05297	97.07103	137.7062	44.19355
	151.2305	53.12529	93.31931	153.2255	28.03725
	234.3374	86.13563	161.3334	97.26199	35.64798
	136.2469	54.52017	134.6881	161.8936	110.1805
	66.54269	114.4598	106.5821	127.7726	49.96534
	78.1216	65.64301	68.55016	133.6048	49.61406
	85.1914	87.50955	131.4279	134.5466	64.04208
	87.85688	96.58521	161.9786	91.22039	42.30333
	84.26753	38.62224	147.8351	72.72278	61.65123
	84.50392	64.61378	149.7902	153.4521	76.57664
	91.89027	50.01869	131.9103	116.3732	30.66712
	121.2959	70.87608	131.2009	128.5877	29.14606
	66.47968	72.67485	132.0147	112.864	33.13901
	142.2906	40.80703	133.6807	92.09046	36.88883
	83.70331	33.78867	106.869	162.1176	41.66231
	90.82279	59.17656	138.8619	152.2658	52.81446
	177.5319	49.29106	112.8039	96.49801	29.26213
	59.03733	60.80285	128.0698	147.8083	29.94403
	187.1883	35.81167	165.3421	118.271	96.33241
	94.9472	54.56751	202.695	161.2585	24.84213
	102.1794	45.80298	86.58716	192.5001	47.4958

74.83517	87.26891	111.9399	154.5578	92.16682
89.20765	72.98554	219.1722	108.0952	91.77303
96.04385	64.33284	189.0577	117.5699	56.44059
71.56194	105.4204	76.1855	129.5374	50.66703
147.2795	51.41284	111.2363	158.4385	80.4588
111.4649	39.40952	170.5822	149.4682	111.4913
	44.75748	177.7681	134.6335	52.8508
	83.72955	156.1429	128.3839	81.6066
		157.4716		83.63766
		116.9993		30.27876
		121.9152		16.41013
		137.5586		73.32921
		189.1178		37.85841
		131.9159		70.01494
		196.259		42.34319
		96.4388		79.70435
		157.1474		89.21072
		141.9516		83.34631
		219.3806		92.13853
		200.7266		83.21544
		166.8277		
		115.928		
		122.5489		
		164.713		
		162.2757		
		204.4326		
		223.0023		
		135.9165		
		137.3991		
		161.0475		
		99.27018		
		94.65791		

194.3645
184.9848

Fig.5f	WT	*dnlg2*
	1.001	0.839734
	1	0.368735
	0.999	0.318476
	1.001	0.512076
	0.999	0.29783

Fig.5h	WT	*dnlg2*
	1.001	0.404463
	1	0.139165
	0.999	0.661959
	1.001	0.082006
	0.999	0.558214

Fig.5i	*C57>Myr-RACK1-HA*	*dnlg2^{KO70}; C57>Myr-RACK1-HA*
	0.071273659	0.181967662
	0.376344086	0.252055387
	0.143340858	0.293832185
	0.105462624	0.316342412
	0.25	0.311726384
	0.139550442	0.210199949
	0.131594906	0.195173582
	0.111012037	0.167036216
	0.073727326	0.144244434
	0.133084876	0.260816327
	0.109425349	0.372559853

0.049338652	0.282079955
0.166234103	0.381434355
0.123017903	0.269064565
0.100292826	0.336448598
0.090535918	0.341960119
0.11501571	0.281799108
0.086980306	0.278353185
0.383597079	0.098685701
0.139395044	0.245751634
0.188446602	0.269751338
0.091446125	0.235867769
0.143594787	0.430631868
0.194887631	0.311838175
0.066051307	0.273092369
0.258889997	0.553133016
0.197691198	0.34525661
0.094698005	0.323104056
0.136638801	0.620631068
0.079409519	0.315929991
0.171928817	
0.112445207	
0.17267525	
0.168990976	
0.177155172	
0.161845311	
0.14190862	
0.318374131	
0.197901049	
0.20084312	
0.156996149	

Fig.5n	WT	RACK1^{RNAi}
	0.9999	0.8473042
	1	0.4988908
	1.0001	0.7069063
	1	0.4241176

Supp. Fig.2c	WT	*dnlg2*
	1.002613	1.009652
	0.994377	1.02423
	1.00301	1.025105
	1.194805	0.70456
	0.843886	0.878711
	0.959729	1.021883
	1.00158	1.150253
	1.251702	0.963436
	0.73125	0.598328
	0.925854	0.897167
	1.091195	1.119605
	1.1433	1.05924
	0.879459	0.935435
	0.963551	0.892409
	1.01369	1.083805
	1.006294	0.967805
	0.993706	0.966141

Supp. Fig.2e	WT	Nlg1^{KO}
	0.986693	0.770748
	0.956892	0.957682
	1.136929	1.106757

	0.919486	0.940534

Supp. Fig.2g	WT	*RACK1*	da>RACK1^{RNAi}
	1	2.479604	1.339003762
	1	1.326623	1.360043486
	1	2.655826	2.351152668
	1	1.562717	1.394297827
	1	1.58664	1.949319077
	1		
