# Supplementary material for: Neuroligin 2 governs synaptic morphology and function through RACK1-cofilin signaling in Drosophila
Source: Commun Biol. 2023 Oct 18;6:1056. doi: 10.1038/s42003-023-05428-3 (PMC10584876; doi:10.1038/s42003-023-05428-3)

Figure 2a

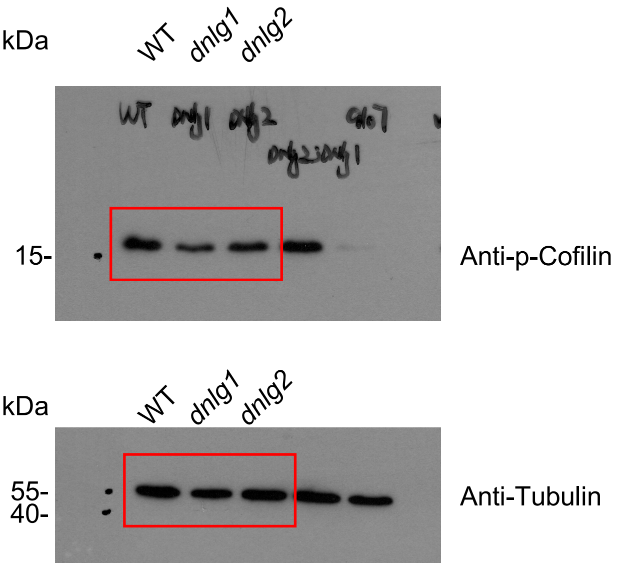

Figure 2c

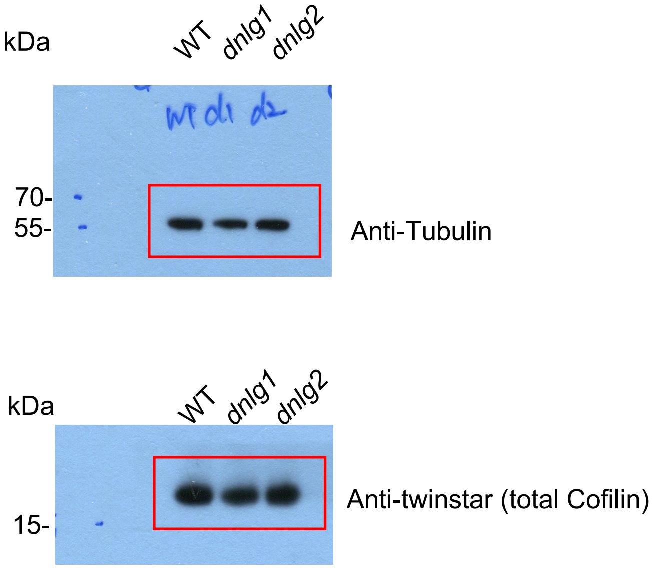

Figure 5a

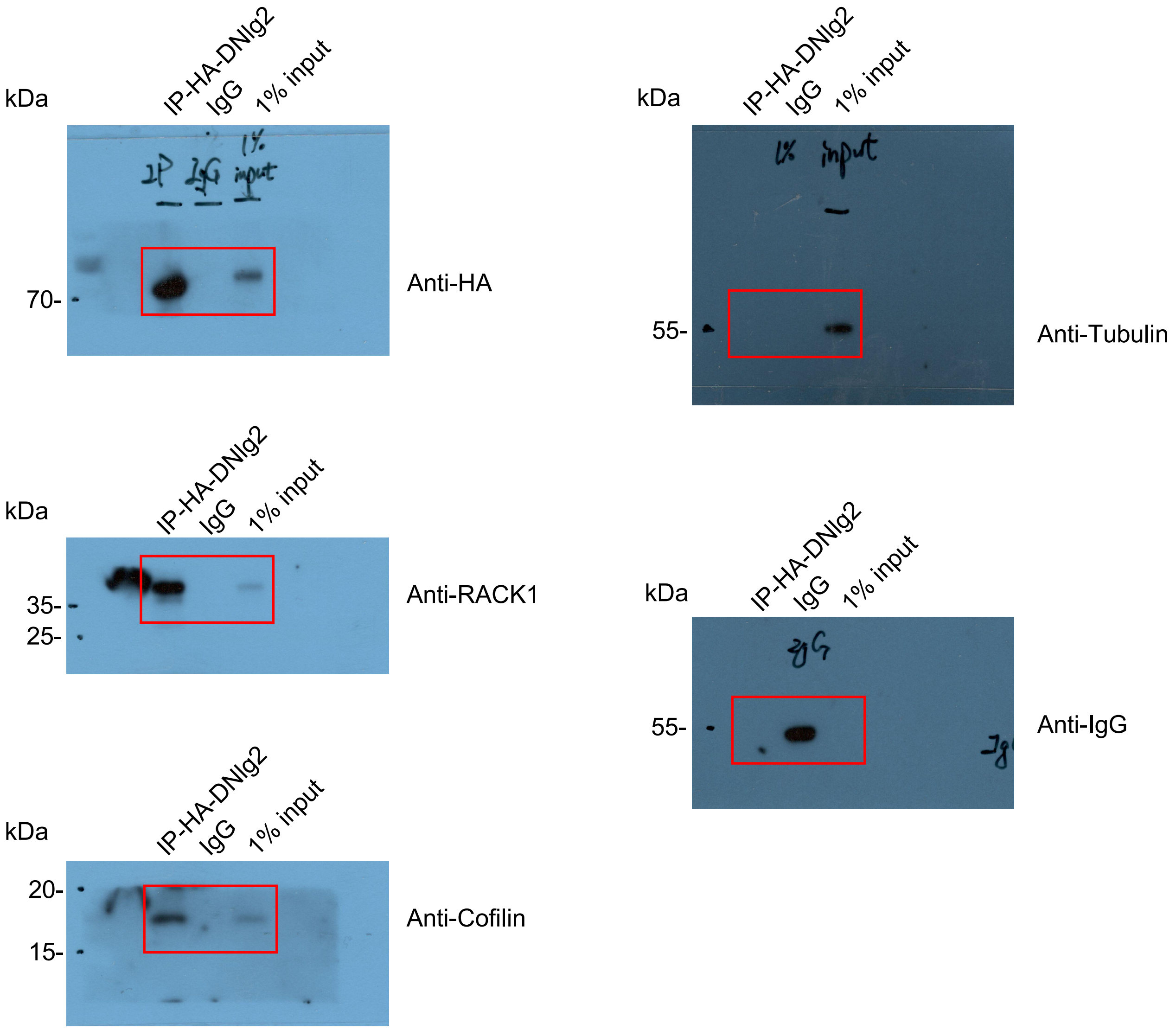
kDa
IP-HA-DNIg2
IgG
1% input
70-
Anti-HA
kDa
IP-HA-DNIg2
IgG
1% input
35-
25-
Anti-RACK1
kDa
IP-HA-DNIg2
IgG
1% input
20-
15-
Anti-Cofilin
kDa
IP-HA-DNIg2
IgG
1% input
55-
Anti-Tubulin
kDa
IP-HA-DNIg2
IgG
1% input
55-
Anti-IgG

Figure 5b

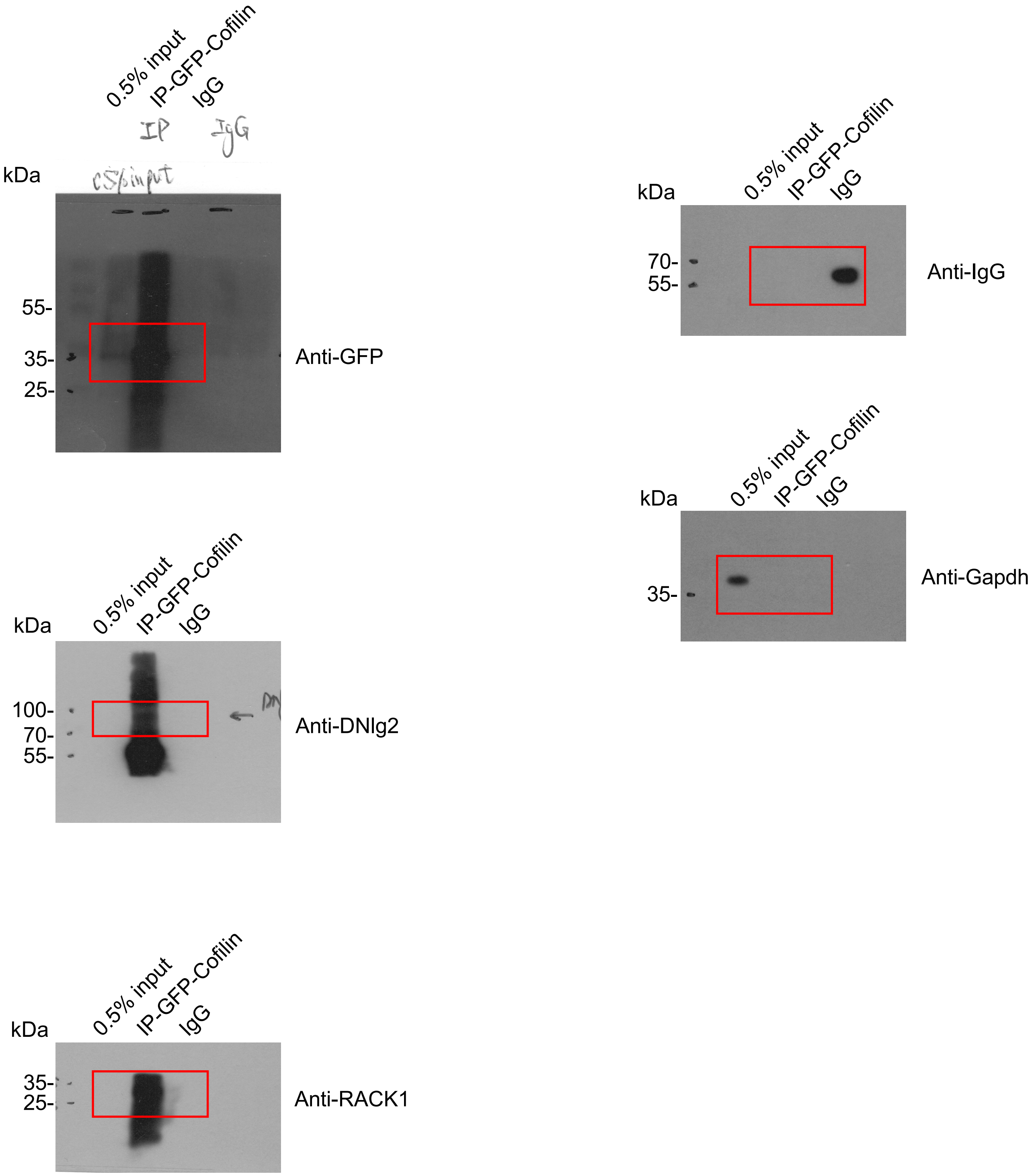
0.5% input
IP-GFP-Cofilin
IgG
kDa
55-
35-
25-
Anti-GFP
0.5% input
IP-GFP-Cofilin
IgG
kDa
70-
55-
Anti-IgG
0.5% input
IP-GFP-Cofilin
IgG
kDa
35-
Anti-Gapdh
0.5% input
IP-GFP-Cofilin
IgG
kDa
100-
70-
55-
Anti-DNlg2
0.5% input
IP-GFP-Cofilin
IgG
kDa
35-
25-
Anti-RACK1

Figure 5d

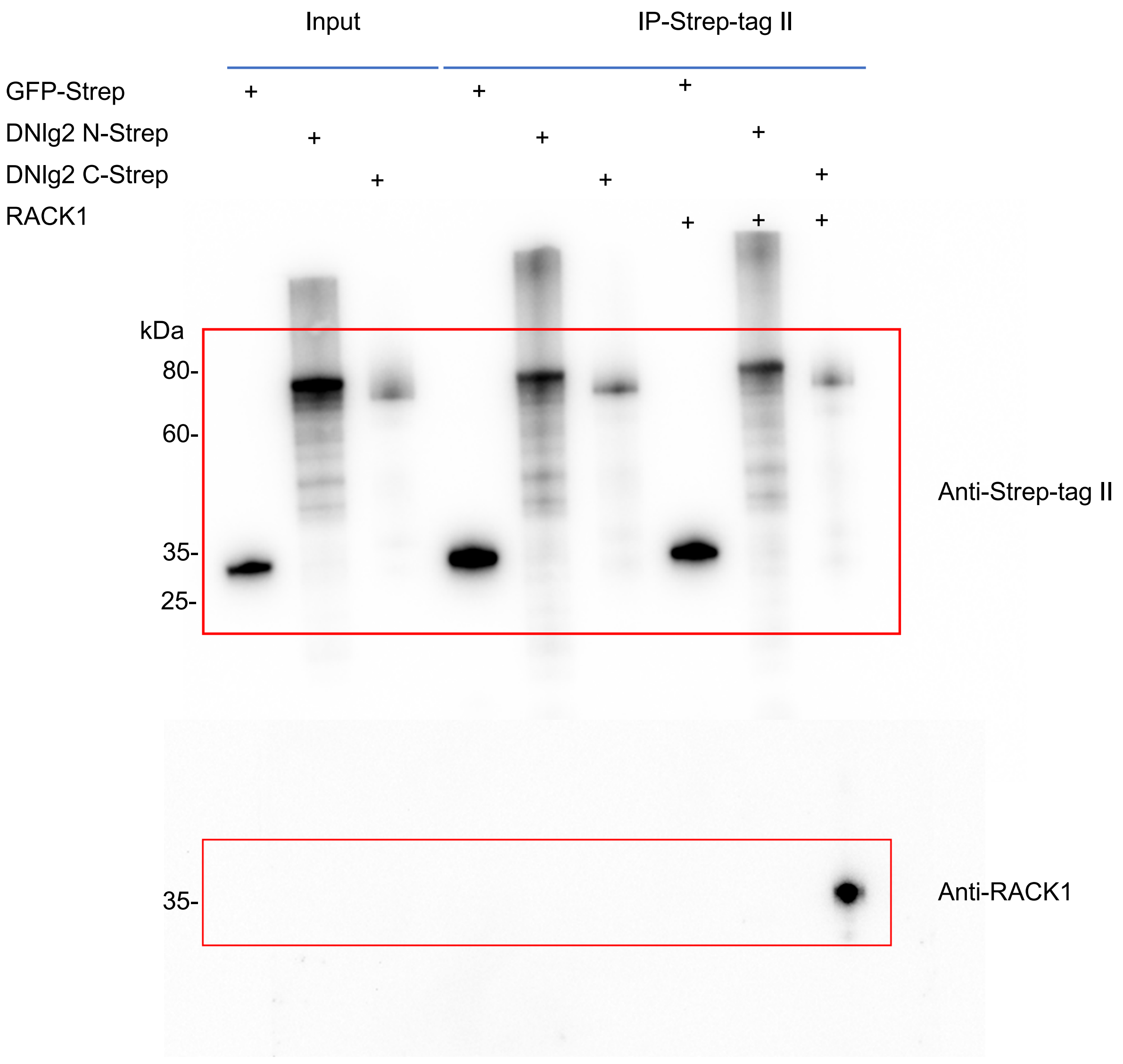

Input
IP-Strep-tag II
GFP-Strep
DNlg2 N-Strep
DNlg2 C-Strep
RACK1
+
kDa
80-
60-
35-
25-
Anti-Strep-tag II
35-
Anti-RACK1

Figure 5e

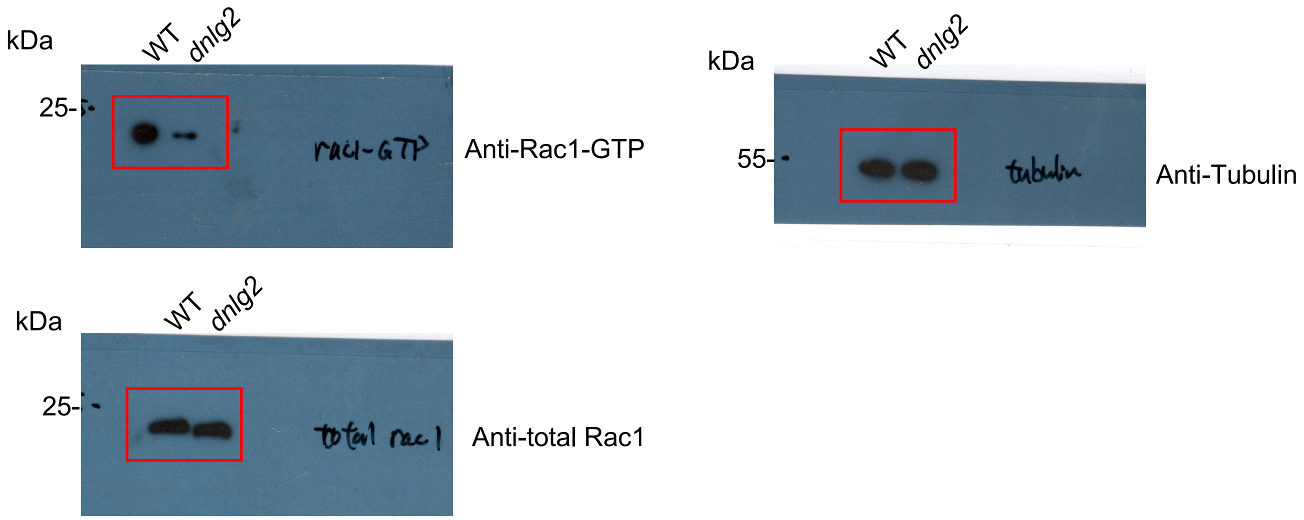

Figure 5g

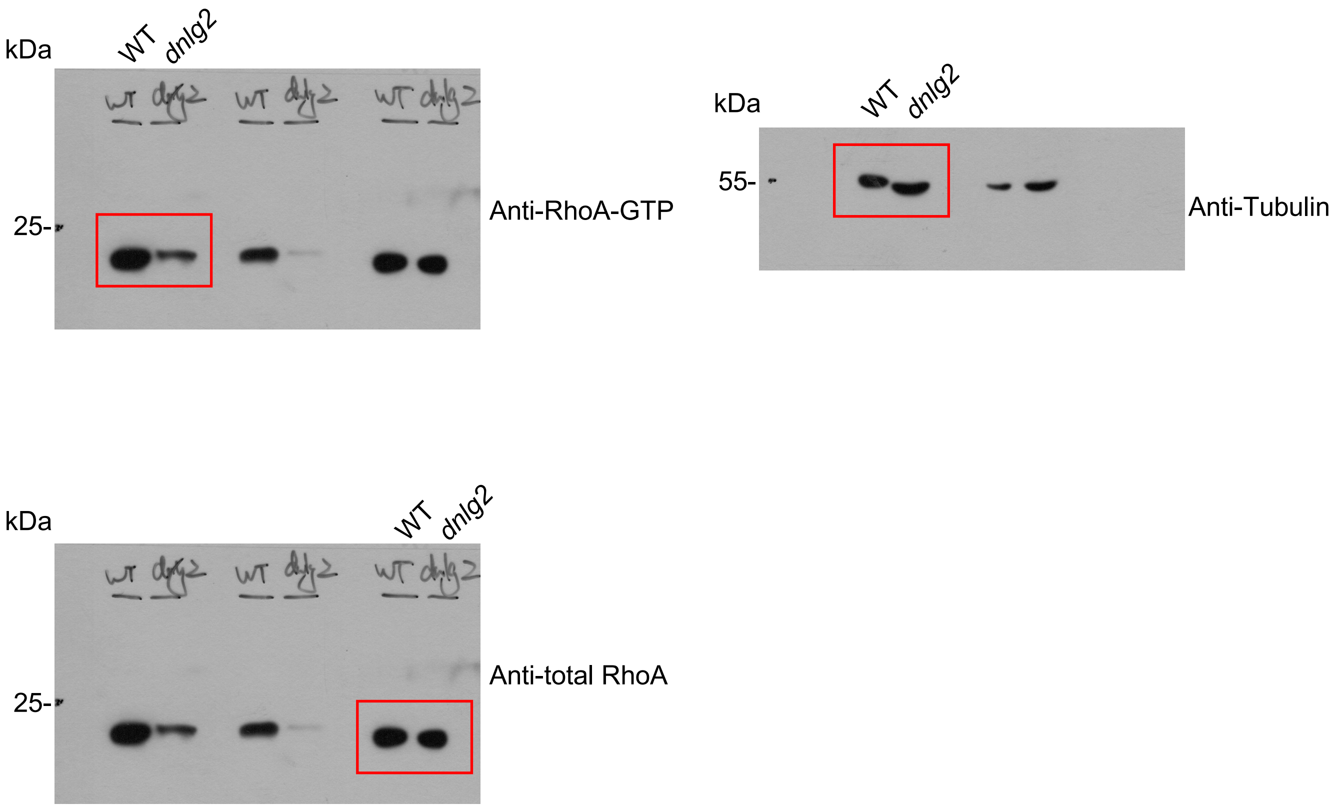

Figure 5i

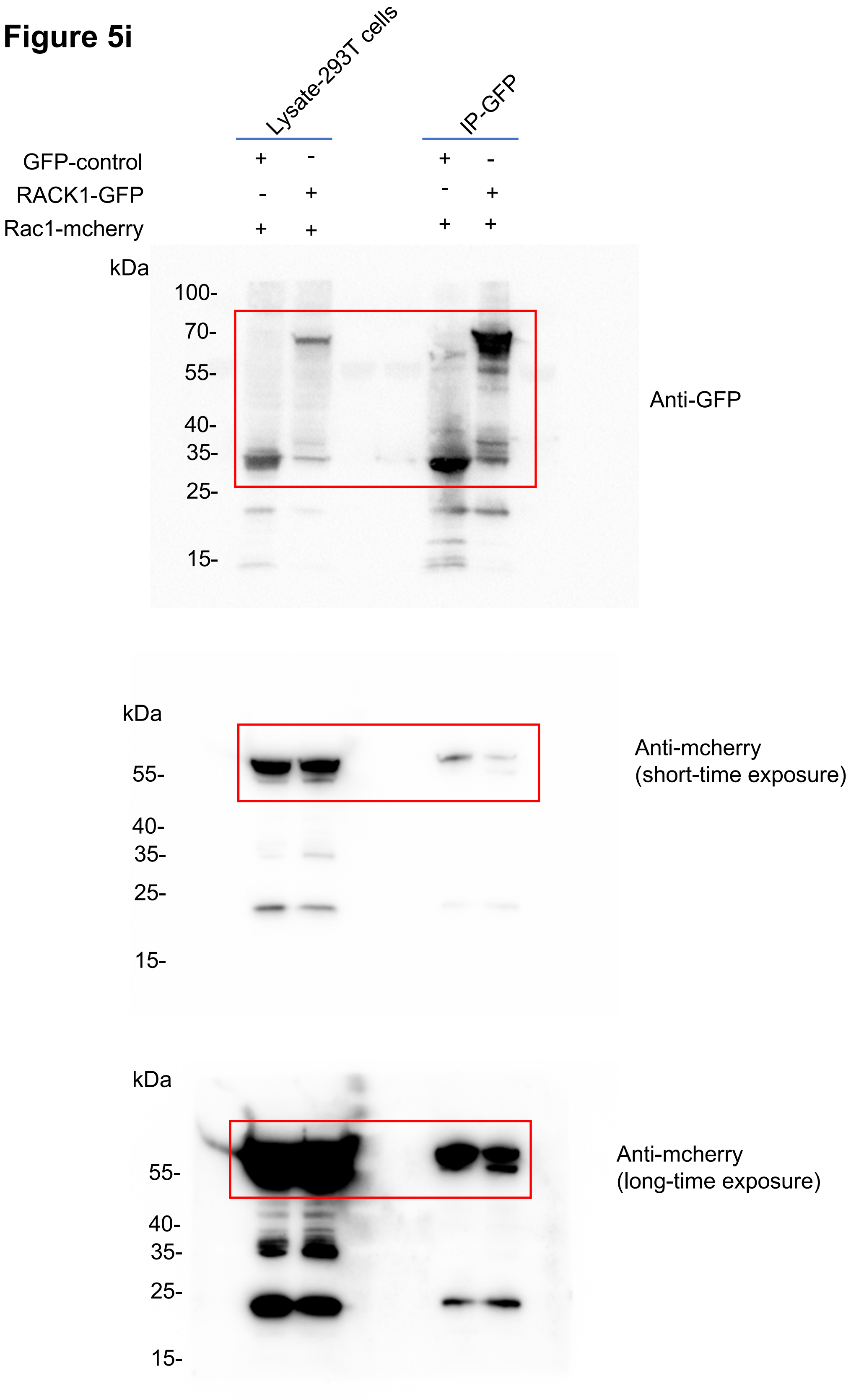

Lysate-293T cells
IP-GFP
GFP-control + - + -
RACK1-GFP - + - +
Rac1-mcherry + + + +
kDa
100-
70-
55-
40-
35-
25-
15-
Anti-GFP
kDa
55-
40-
35-
25-
15-
Anti-mcherry
(short-time exposure)
kDa
55-
40-
35-
25-
15-
Anti-mcherry
(long-time exposure)

Figure 5j

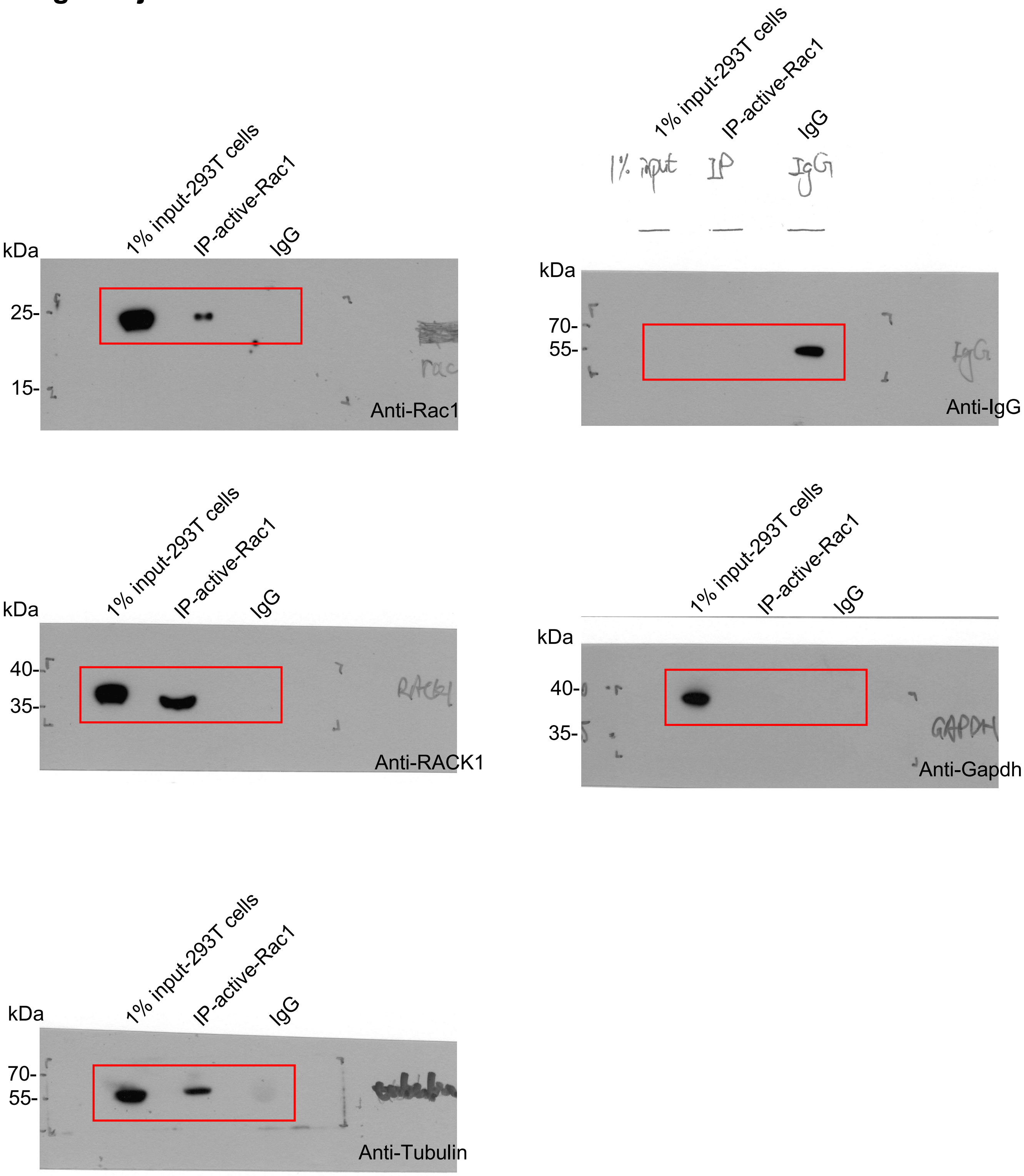
1% input-293T cells
IP-active-Rac1
IgG
kDa
25-
15-
Anti-Rac1
1% input-293T cells
IP-active-Rac1
IgG
kDa
70-
55-
Anti-IgG
1% input-293T cells
IP-active-Rac1
IgG
kDa
40-
35-
Anti-RACK1
1% input-293T cells
IP-active-Rac1
IgG
kDa
40-
35-
Anti-Gapdh
1% input-293T cells
IP-active-Rac1
IgG
kDa
70-
55-
Anti-Tubulin

Figure 5m

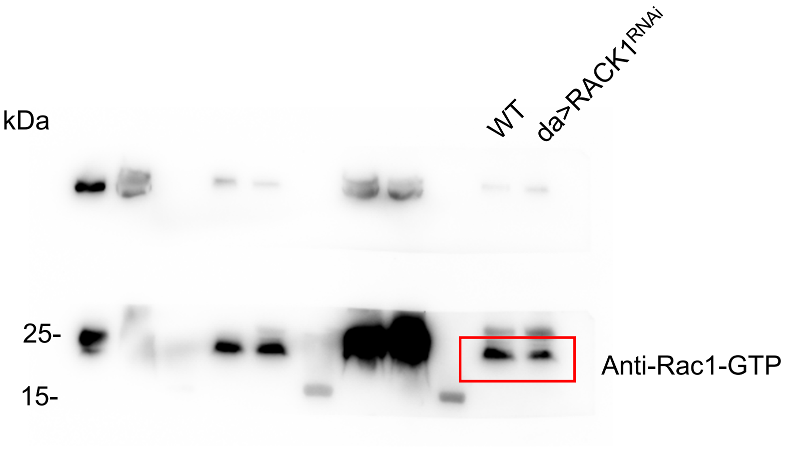

WT
da>RACK1RNAi
kDa
25-
15-
Anti-Rac1-GTP

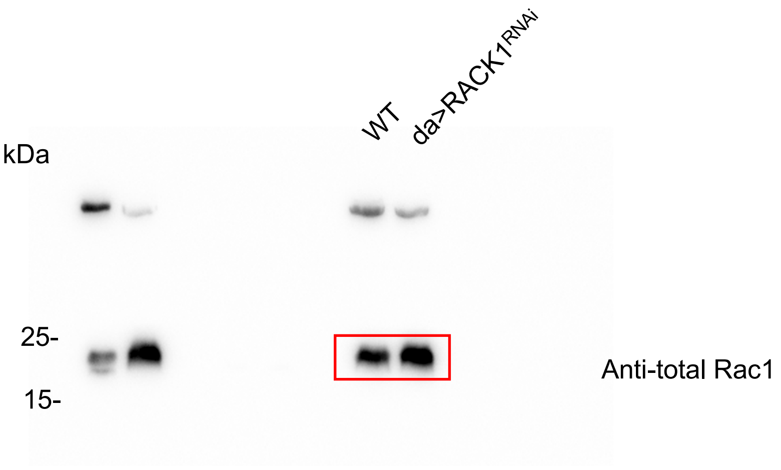

WT
da>RACK1RNAi
kDa
25-
15-
Anti-total Rac1

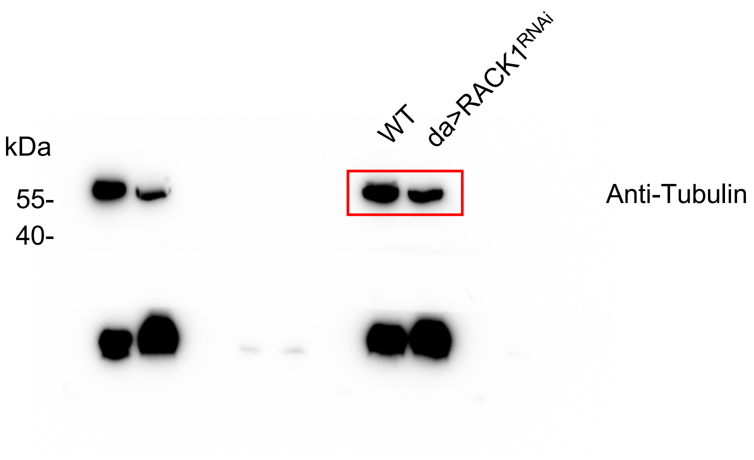

WT
da>RACK1RNAi
kDa
55-
40-
Anti-Tubulin

Supplementary Figure 2a

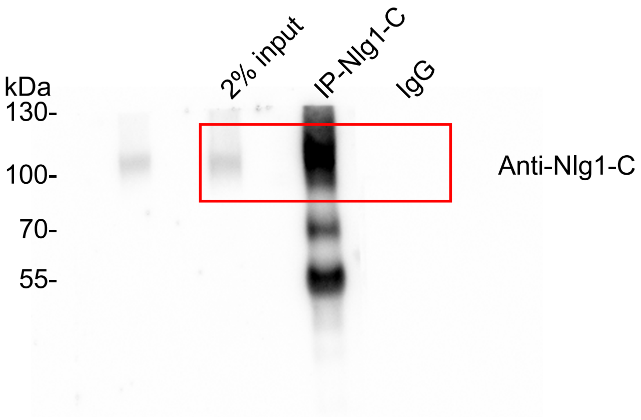

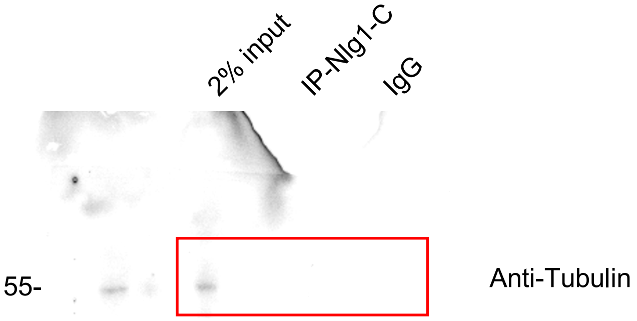

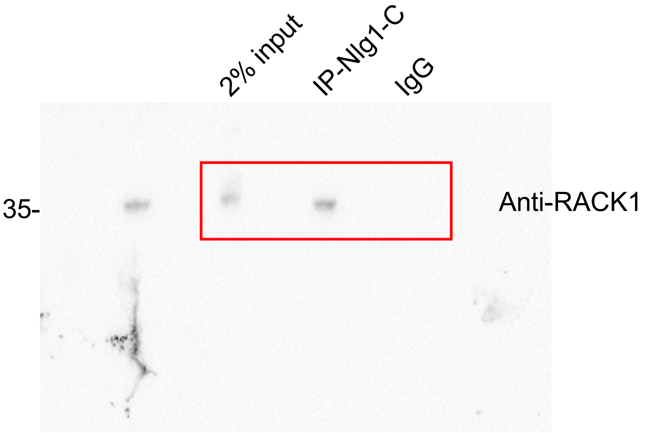

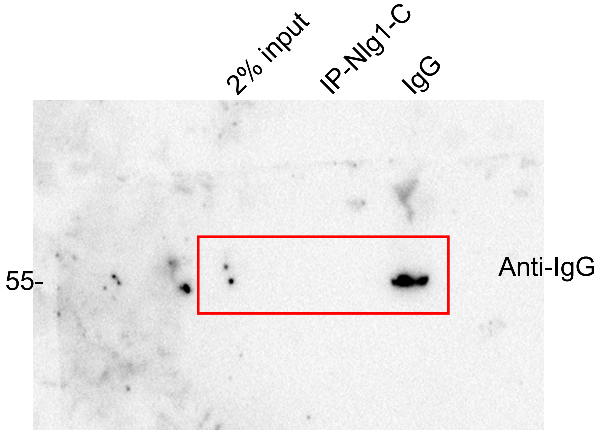

Supplementary Figure 2b

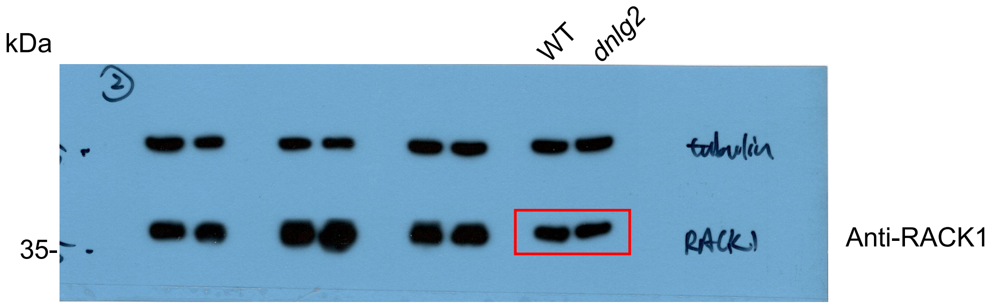

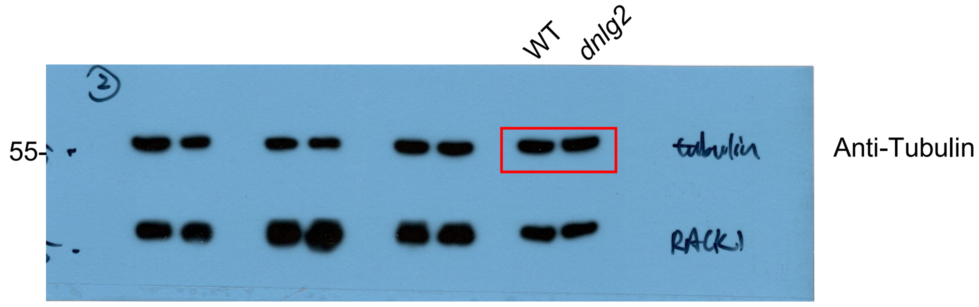

Supplementary Figure 2d

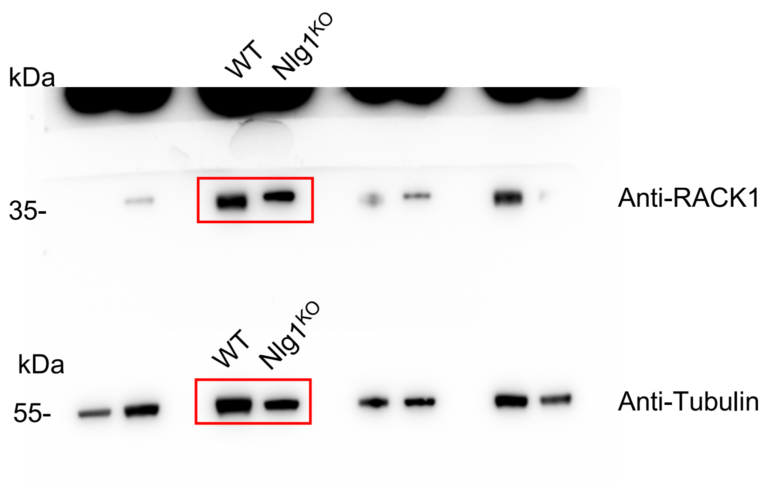

Supplementary Figure 2f

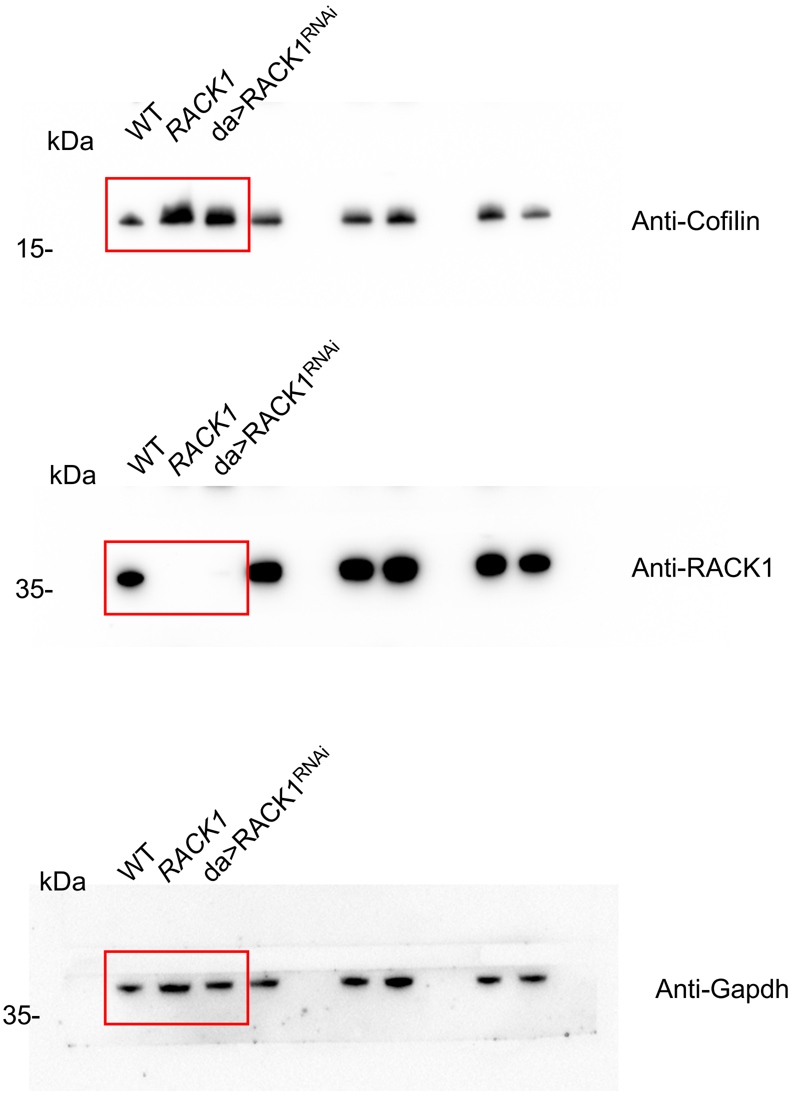

Supplement: Supplementary file 4 — Supplementary data 2 [file 42003_2023_5428_MOESM4_ESM.pdf]
