# Supplementary material for: Neuroligin 2 governs synaptic morphology and function through RACK1-cofilin signaling in Drosophila
Source: Commun Biol. 2023 Oct 18;6:1056. doi: 10.1038/s42003-023-05428-3 (PMC10584876; doi:10.1038/s42003-023-05428-3)
Supplement: Supplementary file 5 — Reporting Summary [file 42003_2023_5428_MOESM5_ESM.pdf]

nature portfolio

Corresponding author(s): Moyi Li, Wei Xie

Last updated by author(s): Sep 25, 2023

Reporting Summary

Nature Portfolio wishes to improve the reproducibility of the work that we publish. This form provides structure for consistency and transparency in reporting. For further information on Nature Portfolio policies, see our Editorial Policies and the Editorial Policy Checklist.

Statistics

For all statistical analyses, confirm that the following items are present in the figure legend, table legend, main text, or Methods section.

n/a	Confirmed	
☐	☒	The exact sample size (*n*) for each experimental group/condition, given as a discrete number and unit of measurement
☐	☒	A statement on whether measurements were taken from distinct samples or whether the same sample was measured repeatedly
☐	☒	The statistical test(s) used AND whether they are one- or two-sided<br>*Only common tests should be described solely by name; describe more complex techniques in the Methods section.*
☒	☐	A description of all covariates tested
☐	☒	A description of any assumptions or corrections, such as tests of normality and adjustment for multiple comparisons
☐	☒	A full description of the statistical parameters including central tendency (e.g. means) or other basic estimates (e.g. regression coefficient) AND variation (e.g. standard deviation) or associated estimates of uncertainty (e.g. confidence intervals)
☐	☒	For null hypothesis testing, the test statistic (e.g. *F*, *t*, *r*) with confidence intervals, effect sizes, degrees of freedom and *P* value noted<br>*Give P values as exact values whenever suitable.*
☒	☐	For Bayesian analysis, information on the choice of priors and Markov chain Monte Carlo settings
☒	☐	For hierarchical and complex designs, identification of the appropriate level for tests and full reporting of outcomes
☒	☐	Estimates of effect sizes (e.g. Cohen's *d*, Pearson's *r*), indicating how they were calculated

Our web collection on statistics for biologists contains articles on many of the points above.

Software and code

Policy information about availability of computer code

Data collection	Microsoft Excel, Image J (1.52),
Data analysis	Microsoft Excel, Image J (1.52), GraphPad Prism (9.0)

For manuscripts utilizing custom algorithms or software that are central to the research but not yet described in published literature, software must be made available to editors and reviewers. We strongly encourage code deposition in a community repository (e.g. GitHub). See the Nature Portfolio guidelines for submitting code & software for further information.

Data

Policy information about availability of data

All manuscripts must include a data availability statement. This statement should provide the following information, where applicable:

- Accession codes, unique identifiers, or web links for publicly available datasets
- A description of any restrictions on data availability
- For clinical datasets or third party data, please ensure that the statement adheres to our policy

All of the relevant data and materials are freely available to any scientist upon request.

Human research participants

Policy information about studies involving human research participants and Sex and Gender in Research.

Reporting on sex and gender	*Use the terms sex (biological attribute) and gender (shaped by social and cultural circumstances) carefully in order to avoid confusing both terms. Indicate if findings apply to only one sex or gender; describe whether sex and gender were considered in study design whether sex and/or gender was determined based on self-reporting or assigned and methods used. Provide in the source data disaggregated sex and gender data where this information has been collected, and consent has been obtained for sharing of individual-level data; provide overall numbers in this Reporting Summary. Please state if this information has not been collected. Report sex- and gender-based analyses where performed, justify reasons for lack of sex- and gender-based analysis.*
Population characteristics	*Describe the covariate-relevant population characteristics of the human research participants (e.g. age, genotypic information, past and current diagnosis and treatment categories). If you filled out the behavioural & social sciences study design questions and have nothing to add here, write "See above."*
Recruitment	*Describe how participants were recruited. Outline any potential self-selection bias or other biases that may be present and how these are likely to impact results.*
Ethics oversight	*Identify the organization(s) that approved the study protocol.*

Note that full information on the approval of the study protocol must also be provided in the manuscript.

Field-specific reporting

Please select the one below that is the best fit for your research. If you are not sure, read the appropriate sections before making your selection.

☒ Life sciences ☐ Behavioural & social sciences ☐ Ecological, evolutionary & environmental sciences

For a reference copy of the document with all sections, see nature.com/documents/nr-reporting-summary-flat.pdf

Life sciences study design

All studies must disclose on these points even when the disclosure is negative.

Sample size	The size for the samples in each experiment varied depending on necessity and the exact size for each experiment was indicated in the figures and figure legends.
Data exclusions	No data were excluded from the analysis.
Replication	All attempts were reliably repeatable. At least three biological replicates were performed for all experiments.
Randomization	Samples were all collected randomly for experiments.
Blinding	Blinding was not possible during experiments as all the samples of different genotypes were performed together, which need to be distinguished. All the samples were analysis together using the same analysis settings.

Reporting for specific materials, systems and methods

We require information from authors about some types of materials, experimental systems and methods used in many studies. Here, indicate whether each material, system or method listed is relevant to your study. If you are not sure if a list item applies to your research, read the appropriate section before selecting a response.

Materials & experimental systems

n/a	Involved in the study	
☐	☒	Antibodies
☐	☒	Eukaryotic cell lines
☒	☐	Palaeontology and archaeology
☐	☒	Animals and other organisms
☒	☐	Clinical data
☒	☐	Dual use research of concern

Methods

n/a	Involved in the study	
☒	☐	ChIP-seq
☒	☐	Flow cytometry
☒	☐	MRI-based neuroimaging

Antibodies

Antibodies used	Primary antibodies against DLG (DSHB:4F3; RRID: AB_528203), BRP (DSHB:NC82; RRID:AB_2314866), GluRIIA (DSHB:8B4D2; RRID:AB_528269), GluRIIB (gifts from Yongqing Zhang, Chinese Academy of Sciences), DNase I (Invitrogen: PA5-76783; RRID: AB_2720510), HRP (Jackson ImmunoResearch:323-005-021 RRID: AB_2314648), p-Cofilin (Santa Cruz: 12912), Cofilin (gifts from James R. Bamburg, Colorado State University), RACK1 (CST: 5432S), HA (Sigma: H9658), GFP (Sigma: G1544), Strep II-Tag (Abconal: AE066), mcherry (Abcam: ab213511) were used.<br>Internal reference protein Tubulin (Sigma: Clone DM1A; RRID: AB_477593), Gapdh (Abcam: ab8245), Rabbit Control IgG (Abconal: AC005; RRID: AB_2771930), Mouse Control IgG (Abconal: AC011; RRID: AB_2770414) were used.<br>Alexa Fluor488-, 555-, or 405-conjugated (Invitrogen) secondary antibodies were used.
Validation	All commercially available antibodies were validated by the suppliers.

Eukaryotic cell lines

Policy information about cell lines and Sex and Gender in Research

Cell line source(s)	human embryonic kidney epithelial-like cell (HEK 293T) (CRL-3216™; ATCC, RRID: CVCL_0063)
Authentication	cell line is not authenticated.
Mycoplasma contamination	Negative
Commonly misidentified lines<br>(See ICLAC register)	No

Animals and other research organisms

Policy information about studies involving animals; ARRIVE guidelines recommended for reporting animal research, and Sex and Gender in Research

Laboratory animals	Drosophila melanogaster, w1118, 3rd stage larvea. Mus Musculus, C57BL/6 and Nlg1KO, 2.5-month-old mice.
Wild animals	This study did not involve wild animals.
Reporting on sex	The information for Drosophila has not been collected. The WB experiment using mice included 2 female mice and 2 male mice.
Field-collected samples	This study did not involve sample collected from the field.
Ethics oversight	All animal procedures were approved by the Laboratory Animal Care and Use Committee of Southeast University, and were performed in strict accordance with the National Institutes of Health Guide for the Care and Use of Laboratory Animals.

Note that full information on the approval of the study protocol must also be provided in the manuscript.
